# Single-cell massively-parallel multiplexed microbial sequencing (M3-seq) identifies rare bacterial populations and profiles phage infection

**DOI:** 10.1038/s41564-023-01462-3

**Published:** 2023-08-31

**Authors:** Bruce Wang, Aaron E. Lin, Jiayi Yuan, Katherine E. Novak, Matthias D. Koch, Ned S. Wingreen, Britt Adamson, Zemer Gitai

**Affiliations:** 1 Lewis-Sigler Institute for Integrative Genomics, Princeton University, Princeton, NJ USA; 2 Department of Molecular Biology, Princeton University, Princeton, NJ USA

**Keywords:** Microbiology, Sequencing

## Abstract

Bacterial populations are highly adaptive. They can respond to stress and survive in shifting environments. How the behaviours of individual bacteria vary during stress, however, is poorly understood. To identify and characterize rare bacterial subpopulations, technologies for single-cell transcriptional profiling have been developed. Existing approaches show some degree of limitation, for example, in terms of number of cells or transcripts that can be profiled. Due in part to these limitations, few conditions have been studied with these tools. Here we develop massively-parallel, multiplexed, microbial sequencing (M3-seq)—a single-cell RNA-sequencing platform for bacteria that pairs combinatorial cell indexing with post hoc rRNA depletion. We show that M3-seq can profile bacterial cells from different species under a range of conditions in single experiments. We then apply M3-seq to hundreds of thousands of cells, revealing rare populations and insights into bet-hedging associated with stress responses and characterizing phage infection.

## Abstract (web summary)

M3-seq uses combinatorial indexing alongside post hoc rRNA depletion in a single-cell RNA sequencing approach that reveals bacterial heterogeneity and rare populations during antibiotic stress and phage infection, as well as bet-hedging responses during growth.

## Main

Bacteria have a remarkable ability to adapt to diverse and changing environments. One strategy that allows populations to flourish in the face of unpredictable environmental stressors is specialization of individual cells. These specializations can manifest as morphological changes (for example, sporulation in Gram-positive organisms)^1^ or visually indistinguishable but functionally distinct states (for example, rare antibiotic-resistant ‘persister’ phenotypes in *Staphylococcus aureus* and *Escherichia coli*)^2–4^. A promising approach to study such specializations is to measure how single cells orchestrate gene expression. For mammalian cells, such measurements have been enabled by single-cell RNA sequencing (scRNA-seq)^5–7^. Despite pioneering efforts to develop similar tools for bacteria, current technologies for studying microbes lag behind.

**Fig. 1:**
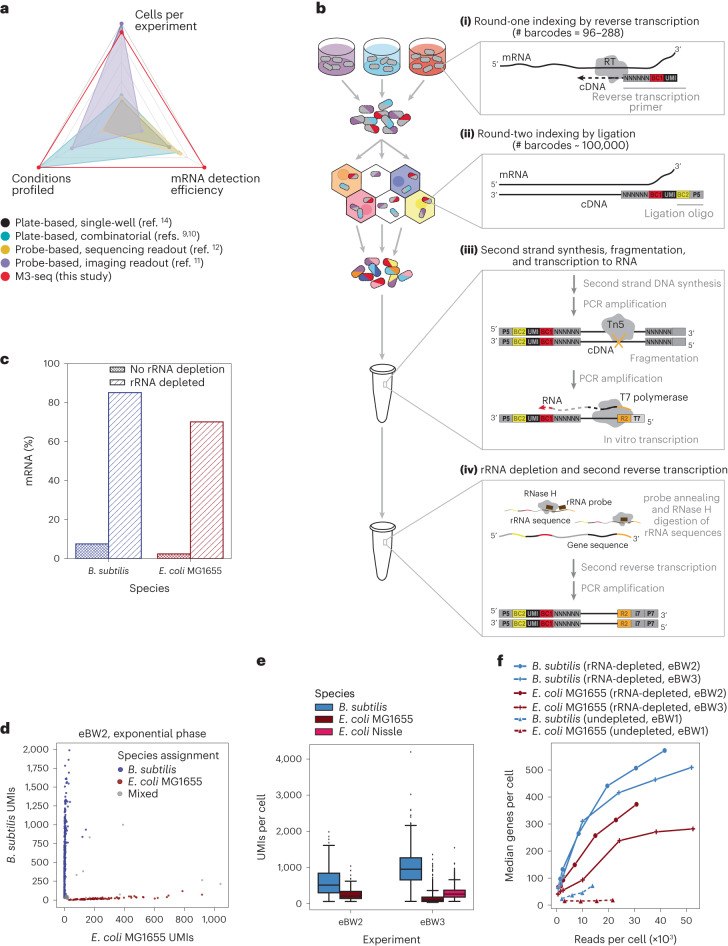
Development of M3-seq platform for single-cell RNA-sequencing with post hoc rRNA depletion. **a**, scRNA-seq methods previously established for bacteria with reported number of cells (ranging from 100 cells per experiment to 300,000 cells per experiment), conditions (ranging from 1 condition per experiment to 20 conditions per experiment) and mRNA genes per cell (ranging from 29 genes per cell to 371 genes per cell). Numbers in each category were selected by taking maximum reported values. Numbers also found in Extended Data Table 1. **b**, Schematic of M3-seq experimental workflow. Indexing: (**i**) RNA molecules are reverse transcribed in situ with indexed primers such that cells in each reaction (that is, separate plate wells) are marked with distinct sequences. Primers allow for random priming. (**ii**) Cells are then collected, mixed and distributed into droplets for a second round of indexing via ligation with barcoded oligos. Sequencing library preparation: Cells are collected again and lysed to release single-strand cDNAs. (**iii**) Second-strand synthesis is then performed in bulk reactions and resulting cDNA molecules are fragmented with Tn5 transposase, amplified via PCR to add a T7 promoter and converted to RNA using T7 RNA polymerase. (**iv**) To deplete ribosomal sequences, the amplified RNA library is hybridized to complementary DNA probes (Supplementary Table 3), and annealed sequences are cleaved by RNase H. Finally, remaining sequences are reverse transcribed back to DNA, sequencing adaptors are added and data are collected by sequencing. **c**, Percentages of mRNA sequences in *B. subtilis* and *E. coli* single-cell libraries prepared with and without rRNA depletion. Data from undepleted libraries come from eBW1 and data from depleted libraries come from eBW3. **d**, M3-seq analysis of a mixture of *B. subtilis* and *E. coli* wherein each point corresponds to a single ‘cell’ (that is, unique combination of plate and droplet barcodes). Species assignments were made as described in Methods. **e**, UMIs per cell (after species assignment) observed in exponential-phase cells across two experiments, eBW2 and eBW3 (515 ± 245 and 953 ± 310 median UMIs with absolute deviation for *B. subtilis*, respectively; 211 ± 85 and 100 ± 47 median UMIs with absolute deviation for *E. coli* MG1655, respectively; 266 ± 100 UMIs with for *E. coli* Nissle in eBW3). *N* = 1,336, 533, 84, 1,944, 1,659 cells, respectively. Boxplot limits are as defined in Methods. **f**, Median genes detected per *B. subtilis* or *E. coli* cell as a function of the number of total reads per cell across three experiments: eBW1, eBW2 and eBW3.

Existing bacterial scRNA-seq methods include MATQ-seq^8^, PETRI-seq^9^, microSPLiT^10^, par-SeqFISH^11^ and ProBac-seq^12^ (Fig. 1a and Extended Data Table 1). Each of these methods uses a different strategy to index cells and their transcripts, and each has benefits and drawbacks^13^. MATQ-seq isolates single cells into separate wells of multiwell plates and performs individual indexing reactions to generate sequencing libraries^14^. This ‘indexing’ scheme is inherently limited in scale. By contrast, each of the remaining methods allows single-cell gene expression to be profiled across pools of cells in single experiments, with multiplexed transcript detection enabled by in situ probe hybridization (SeqFISH and ProBac-seq) or split-pool combinatorial indexing^7^ (PETRI-seq, microSPLiT). These methods have established the field of single-cell transcriptomics in bacteria, but drawbacks remain. Hybridization-based approaches rely on pre-designed species- and gene-specific probes, thus limiting unbiased discovery, while combinatorial indexing platforms have an abundance of signal from ribosomal (r)RNA, which can compromise messenger (m)RNA detection. Given these considerations, here we develop massively-parallel, multiplexed, microbial sequencing (M3-seq), a method for scRNA-seq in bacteria that combines plate-based, in situ indexing with droplet-based indexing and post hoc rRNA depletion. In parallel to our study, another droplet-based, scRNA-seq method, called BacDrop^15^, was reported. This method performs rRNA depletion in situ^15^, while M3-seq performs rRNA depletion after library amplification, thus reducing the risk of losing unamplified, non-rRNA transcripts and potentially increasing sensitivity. M3-seq enables massively parallel gene expression profiling of single bacterial cells across many samples at transcriptome-scale with sensitive mRNA capture. By applying M3-seq to hundreds of thousands of cells, we revealed independent phage induction programmes in *Bacillus subtilis*, a bet-hedging subpopulation of *E. coli* and the detailed heterogeneity of phage infection.

## Results

### M3-seq captures rRNA-depleted single-cell transcriptomes

**Extended Data Fig. 1:**
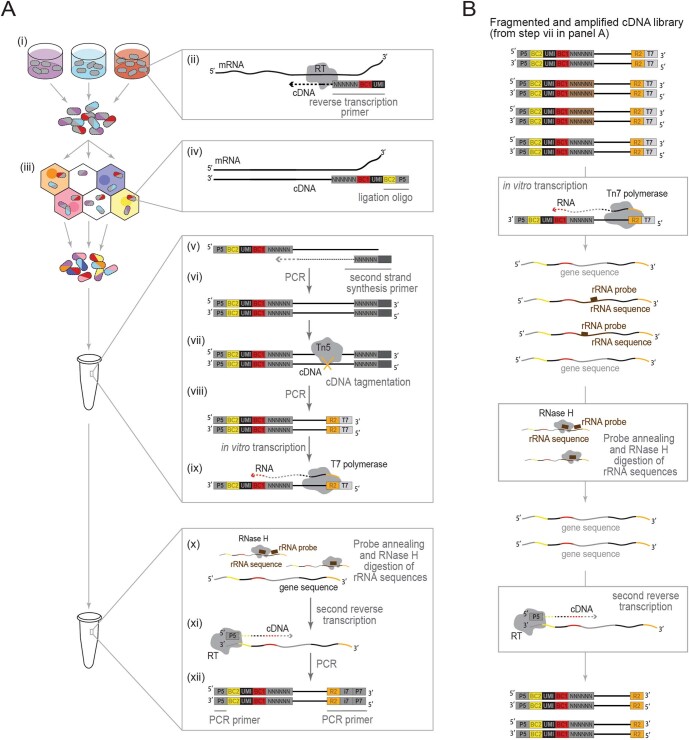
M3-seq experimental workflow and rRNA depletion scheme. **a**. Detailed schematic of M3-seq experimental workflow: Populations of fixed and permeabilized bacteria are (i) aliquoted into wells of one or more 96 well plates. Each well contains a uniquely indexed random hexamer, which acts as a primer for (ii) *in situ* reverse transcription. These primers also carry unique molecular identifier (UMIs) sequences. During reverse transcription, cell-associated RNA molecules are converted to cDNAs with primer barcodes and UMIs on their 5’ ends. After reverse transcription, (iii) cells are pooled and loaded into a commercially available device for droplet-based indexing (herein, the Chromium Controller from 10x Genomics) without a need for limiting dilution. After partitioning into droplets, (iv) a second index is ligated onto the 5’ end of the reverse transcribed, cell-associated cDNAs (herein, using Next GEM Single Cell ATAC reagents from 10x Genomics). Following indexing, cells are lysed, and (v) cDNA molecules are converted to double-strand DNA using a Klenow enzyme and a random primer with a PCR handle at the 5’ end. This double-strand cDNA is then (vi) amplified by PCR, (vii) fragmented with Tn5 transposase loaded with Nextera read 2 primers, and (viii) attached to a T7 promoter via a second round of PCR. Next, (ix) cDNA molecules are transcribed back into RNA using T7 RNA polymerase. This step prepares the amplified library for rRNA depletion. After transcription, (x) the resulting RNA is annealed to a set of DNA probes that are complementary to rRNA sequences within the library (Supplementary Table 4). This annealing allows for selective degradation of those sequences with RNase H. Finally, in a second reverse transcription step, (xi) the indexed and rRNA-depleted library is converted back into cDNA, and (xii) the resulting cDNA is amplified one more time to add a required sequencing adaptor. The library is then ready for paired-end sequencing. **b**. Detailed schematic of rRNA depletion steps: To remove rRNA sequences from M3-seq libraries, we (i) convert indexed and amplified cDNA libraries into RNA via *in vitro* transcription, (ii) hybridized rRNA sequences within the library to DNA probes and digest those sequences using RNase H, and (iii) convert the remaining sequences back into DNA using a P5 primer.

**Extended Data Fig. 2:**
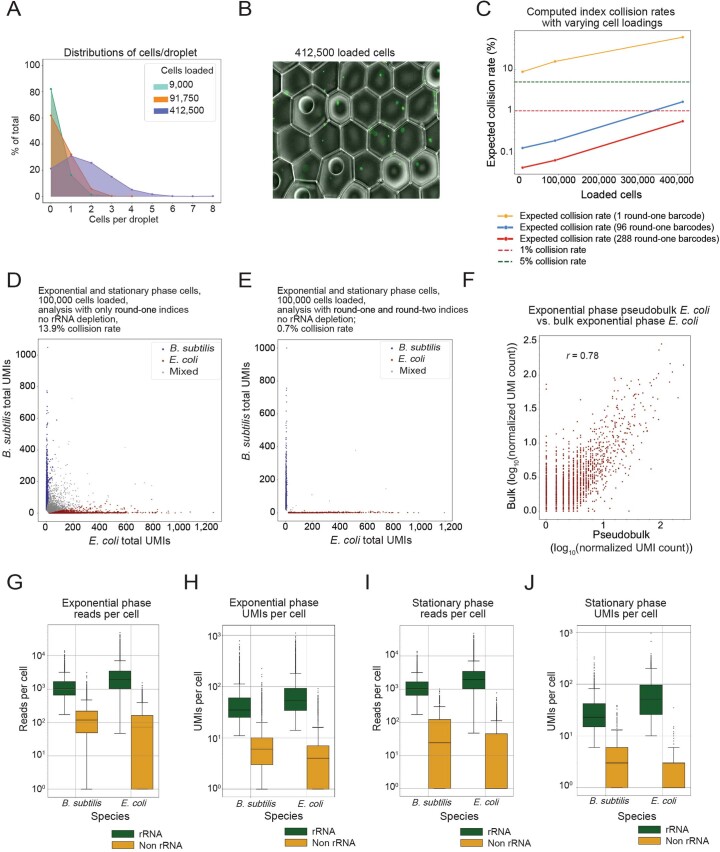
Piloting single-cell RNA-sequencing in bacteria without rRNA depletion. **a**. Distributions represent bacterial cells per droplet produced on the Chromium Controller (10x Genomics) at indicated cell loading numbers. Bacterial droplet loading was quantified as described in *Materials and Methods*. **b**. Representative image of droplets quantified in panel (A). *E. coli* cells, which were stained with Sytox green, are visible as green dots. **c**. Curves represent expected index collision rates (that is, percentage of cells with the same round-one index labelled with the same round-two index) as a function of loaded cells using different indexing schemes. **d**. Analysis of a mixture of exponential and stationary phase *B. subtilis* (blue) and *E. coli* (red) using only round-two (droplet-based) indexes. Species assignments for each ‘cell’ were made as determined in *Materials and Methods*. Data were generated without rRNA depletion (eBW1 in Supplementary Table 2). **e**. Same as (D) but analysis performed with combinatorial barcodes. **f**. Comparison of bulk RNA-seq data to pseudobulk, computationally rRNA-depleted single-cell gene expression in exponential phase *E. coli* from eBW1. Each point represents a single gene. *r*, Pearson correlation. **g**. Read counts per cell from single-cell gene expression data without rRNA depletion (1023 ± 436 reads for *B. subtilis* and 1926 ± 1054 reads for *E. coli* when considering rRNAs; 117 ± 81 reads for *B. subtilis* and 72 ± 72 reads for *E. coli* when considering only non-rRNAs). Data was collected from a single experiment; over *B. subtilis* 4601 cells and *E. coli* 5883 cells. Boxplot limits are as defined in *Materials and Methods*. **h**. Same as (G) but for UMI counts per cell (34 ± 13 UMIs for *B. subtilis* and 52 ± 23 UMIs for *E. coli* when considering rRNAs; 5 ± 5 UMIs for *B. subtilis* and 3 ± 3 UMIs for *E. coli* when considering only non-rRNAs). **i**. Same as (G) but for stationary phase *B. subtilis* (1838 cells) and *E. coli* (2094 cells) (720 ± 380 reads for *B. subtilis* and 1902 ± 1297 reads for *E. coli* when considering rRNAs; 23 ± 23 reads for *B. subtilis* and 0 ± 0 reads for *E. coli* when considering only non-rRNAs). **j**. Same as (H) but for stationary phase *B. subtilis* and *E. coli* (22 ± 10 UMIs for *B. subtilis* and 50 ± 30 UMIs for *E. coli* when considering rRNAs; 2 ± 2 UMIs for *B. subtilis*, and 0 ± 0 UMIs for *E. coli* when considering only non-rRNAs). Data above are reported as medians with maximum average deviation.

We designed M3-seq with two rounds of cell indexing (Fig. 1b and Extended Data Fig. 1). The first of these indexing rounds uses in situ reverse transcription with random priming to tag transcript sequences with one cell index (BC1) and a unique molecular identifier (UMI). This indexing step, which we refer to as ‘round-one indexing’, occurs in multiple reactions, each performed on a separate pool of fixed, permeabilized bacterial cells. After this step, cells are mixed and then separated again into droplets using a commercially available kit (Chromium Next GEM Single Cell ATAC, 10X Genomics). In these droplets, we perform ‘round-two indexing’, wherein a second cell index (BC2) is ligated onto cell-associated, BC1-indexed complementary (c)DNA molecules. While neither BC1 nor BC2 are necessarily unique, together these sequences create a combinatorial index that serves as a distinct marker for individual cells. Conceptually, this indexing scheme is identical to scifi-RNA-seq^16^, which has enabled sequencing of >100,000 mammalian cells in a single run. However, because bacteria are considerably different from mammalian cells (for example, smaller, thick cell walls), we first performed a series of pilot experiments. First, to verify that we could load single-cell suspensions of bacterial cells into droplets at rates appropriate for combinatorial indexing, we loaded different numbers of Sytox Green-stained *E. coli* into droplets and calculated the distribution of cells within resulting droplets by imaging (Extended Data Fig. 2a,b). We then calculated the rates at which cells with the same round-one index would be expected to acquire an identical round-two index (Extended Data Fig. 2c). We call such events ‘index collisions’. With ~96 round-one indices, our calculations suggest that hundreds of thousands of cells can be loaded in a single run of the droplet system with <1% collision rate.

Next, we verified that even though bacterial cells are surrounded by thick cell walls and contain very few mRNA molecules, we could generate single-cell transcriptomes using our approach. Briefly, after growing both *B. subtilis* 168 and *E. coli* MG1655 separately to exponential and stationary phase, we fixed, washed and permeabilized the cells with lysozyme^9,10^. We then combined the cells at equal cell numbers, performed combinatorial indexing using 96 round-one indices and loaded 100,000 cells into droplets for round-two indexing (1 channel of a Single Cell ATAC chip). We refer to this experiment as eBW1 (Supplementary Tables 1 and 3). Given our previous loading calculations, we would expect 15.7% of all cell-containing droplets in this experiment to yield an index collision without round-one indexing. Similar to these expectations, our data revealed a 13.9% collision rate (fraction of cells with <85% of UMIs assigned to one species) between *B. subtilis* and *E. coli* cells when only BC2 indices were used to discriminate cells (Extended Data Fig. 2d). To account for within-species collisions that would otherwise be identified as single *E. coli* or *B. subtilis* cells, we scale this collision rate by a factor of $\frac{1}{2{pq}}$, where *p* is the fraction of *E. coli* cells in the dataset and *q* is the fraction of *B. subtilis* cells in the dataset, such that *p* + *q* = 1. Using this scaling factor gives a total 28.7% collision rate when also accounting for within-species collisions. Encouragingly, using both BC1 and BC2 indices dramatically decreased this rate to 0.7% (1.5% when accounting for within-species collisions) (Extended Data Fig. 2e). In addition, pseudobulk libraries generated from these data have profiles similar to that of bulk RNA-seq (Extended Data Fig. 2f).

**Extended Data Fig. 3:**
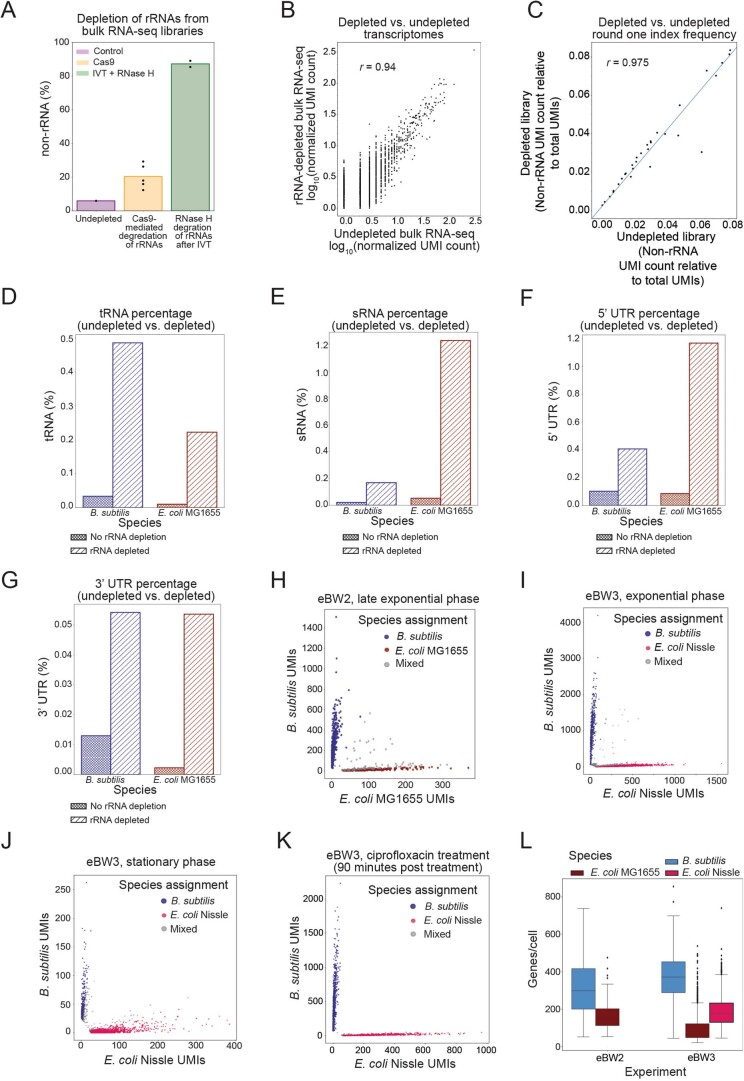
Additional analysis of M3-seq development. **a**. Efficiency of rRNA depletion using two different *post hoc* approaches: degradation by rRNA-targeted Cas9^17,18^ (yellow) and RNase H-mediated digestion after *in vitro* transcription (green). **b**. Comparison of gene expression data from rRNA-depleted and control libraries. Bulk libraries were prepared as in *Materials and Methods*. *r*, Pearson correlation. **c**. Comparison of 30 round-one barcode frequencies from an RNA-seq library before and after *post hoc* rRNA depletion. Bulk libraries were prepared and depleted of rRNA as in *Materials and Methods*. *r*, Pearson correlation. **d**. Percentages of tRNA sequences in *B. subtilis* and *E. coli* single-cell libraries prepared with and without rRNA depletion. Data from undepleted libraries come from eBW1, and data from depleted libraries come from eBW3. **e**. Same as (D) but for sRNAs. **f**. Same as (D) but for 5’ UTRs. **g**. Same as (D) but for 3’ UTRs. **h**. M3-seq analysis of a mixture of *B. subtilis* (blue) and *E. coli* (red) in late exponential phase (OD = 2.1, 2.0 respectively) wherein each point corresponds to a single ‘cell’. Species assignments as described in *Materials and Methods*. We observed a 13% collision rate, 30% corrected to include same-species collisions. Data were generated with rRNA depletion (eBW2 in Supplementary Table 2). **i**. Same as (H) but for *B. subtilis* and a different strain of *E. coli* (OD = 0.3, 0.3 respectively). Data were generated with rRNA depletion (eBW3 in Supplementary Table 2) and show a 12% collision rate, 32% corrected. **j**. Same as (I) but for cells in early stationary phase (OD = 2.4, 3.0 respectively). Data were generated with rRNA depletion (eBW3 in Supplementary Table 2) and show a 6.1% collision rate, 22% corrected. **k**. Same as (I) but for cells 90 minutes post ciprofloxacin treatment (eBW3 in Supplementary Table 2). Data show a 1.84% collision rate, 3.68% corrected, **l**. Genes per cell (after species assignment) observed in exponential phase cells across two experiments, eBW2 and eBW3 (298 ± 104 and 371 ± 82 median genes with absolute deviation for *B. subtilis*, respectively; 151 ± 47 and 75 ± 31 median genes with absolute deviation for *E. coli* MG1655, respectively; 175 ± 50 genes with for *E. coli* Nissle in eBW3). Boxplot limits are as defined in *Materials and Methods*.

As has been previously observed with other bacterial combinatorial indexing methods^9,10^, most reads in our pilot experiment aligned to rRNAs (Extended Data Fig. 2g–j). Of roughly 1,000–2,000 reads per cell in exponential-phase *E. coli*, 90–97% of the reads aligned to rRNA, while the rest aligned to other RNA species (for example, mRNA, transfer (t)RNAs, small(s)RNAs and 5’ or 3’ untranslated regions (UTRs)). Technically, higher coverage of the latter set of RNAs could be achieved by sequencing to greater depth, but we sought a more cost-effective solution removing rRNA sequences before sequencing. When developing this solution, we noted that depletion of rRNA in situ can decrease mRNA capture efficiency^10^ and thus focused on depleting rRNAs after amplification (Fig. 1b and Extended Data Fig. 1a). Specifically, after testing two approaches for depleting ribosomal sequences from bulk libraries (Extended Data Fig. 3a–c), we chose an RNase H-based approach^17–19^ to complete our pipeline (Extended Data Fig. 1b). Our full M3-seq pipeline is as follows: After two rounds of indexing (performed as described above), cDNA libraries are transcribed to single-stranded RNA. rRNA sequences within the library are then hybridized to rRNA-specific DNA probes and digested with RNase H, which specifically cleaves RNA in RNA:DNA hybrids. The resulting rRNA-depleted libraries are then reverse transcribed back into cDNA for sequencing. Encouragingly, putting these steps together enabled recovery of single-cell transcriptomes with an 11–27-fold increase in reads aligning to mRNA (Fig. 1c), a 15–20-fold increase in tRNA (Extended Data Fig. 3d), an 8–21-fold increase in sRNAs (Extended Data Fig. 3e) and a 5–20-fold increase in 5’ and 3’ UTRs (Extended Data Fig. 3f,g) compared with undepleted libraries obtained in eBW1. In addition, the mRNA content of our rRNA-depleted bulk libraries was similar to libraries that had not been depleted (*r* = 0.94) (Extended Data Fig. 3b) and the frequency of individual indices was similar before and after depletion (Extended Data Fig. 3c), implying that the depletion process does not meaningfully change library composition.

To evaluate the full M3-seq pipeline in terms of UMI capture, single-cell resolution and information captured across different conditions, we next performed two large experiments (Supplementary Tables 1 and 3): one in which we evaluated *B. subtilis* 168 and *E. coli* MG1655 (eBW2) and one in which we evaluated these species alongside a non-domesticated strain of *E. coli* (Nissle 1917, eBW3). In these experiments, we grew bacteria to exponential (optical density (OD) = 0.3) and early stationary phases (OD = 2.5, 2.8 and 2.6) with and without antibiotic treatments. After in-plate, round-one indexing, we pooled cells from each condition and loaded them into droplets (Supplementary Table 2). Consistent with our previous experiments, we observed a low index collision rate among cells loaded into droplets (Fig. 1d, Extended Data Fig. 3h–k), although collision rates for these particular libraries were variable across the different treatments and moderately higher than observed in our previous experiments (1.7%–13% collision rate, 3.6%–32% corrected) (Extended Data Fig. 2e, 3h-k).

**Extended Data Fig. 4:**
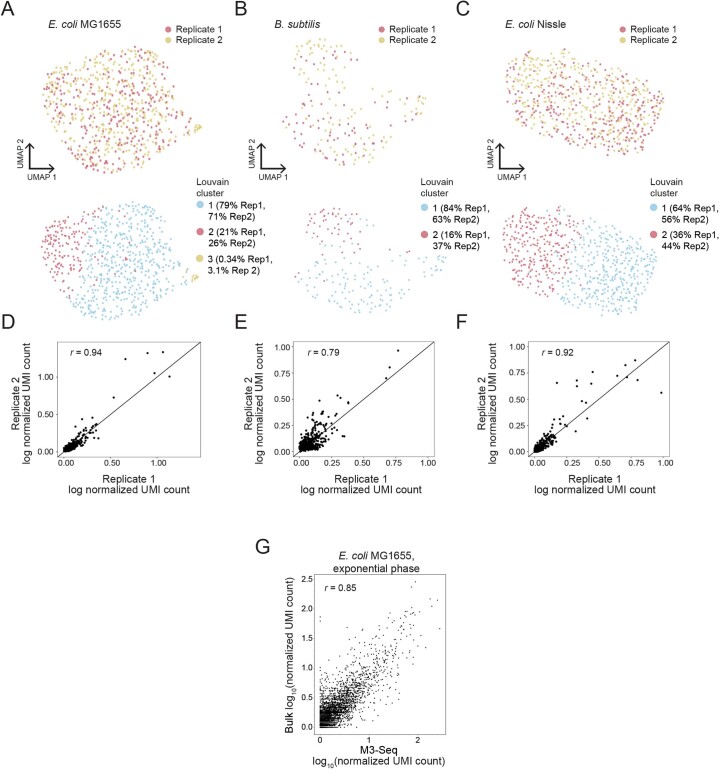
M3-seq profiling during exponential growth and early stationary phase. **a**. Top: UMAP of replicate M3-seq data generated from *E. coli* MG1655 treated with twice the minimum inhibitory concentration of ciprofloxacin, sampled after 6 hours of treatment. **b**. Same as (A) but for *B. subtilis* 168. **c**. Same as (A) but for *E. coli* Nissle. **d**. Comparison of replicate data from (A) using mean log normalized UMI counts per cell (that is, unique UMIs relative to total UMIs per cell averaged across all cells for each gene). Each point represents a single gene. *r*, Pearson correlation. **e**. Same as (D) but using data from (B). **f**. Same as (D) but using data from (C). **g**. Comparison of RNA-seq data to M3-seq pseudobulk profiles from exponential phase *E. coli* from eBW3. Pseudobulk measurements were obtained by normalizing UMI counts by the total number of UMIs in the dataset and log transforming the normalized counts. Each point represents a single gene. *r*, Pearson correlation.

After identifying single cells using combined round-one and round-two indices, we discriminated samples by round-one indices and identified species using the aligned mRNA transcripts. Across two independent experiments (eBW2, eBW3), we recovered 515 and 984 median UMIs per exponential-phase *B. subtilis* cell (298 and 371 median genes per cell, 0.145 and 0.237 mean UMIs per gene), 211 and 100 median UMIs per exponential-phase *E. coli* MG1655 cell (151 and 72 median genes per cell, 0.0654 and 0.0374 UMIs per gene) and 266 median UMIs per exponential-phase Nissle cell (175 median genes per cell), respectively (Fig. 1e and Extended Data Fig. 3l). Compared to other studies that applied scRNA-seq to bacteria, this represents roughly the same number of UMIs per cell^9,10,12^ and UMIs per gene for *E. coli*^9,12^ but twice as many UMIs per cell^10,12^ and UMIs per gene for *B. subtilis*^12^. We found that biological replicates of *E. coli* MG1655, *B. subtilis* 168, and *E. coli* Nissle after 6 hours of drug treatment had similar compositions (Extended Data Fig. 4a–c) and correlated biological signal between replicates (Pearson correlation of (*r* = 0.94, 0.79, 0.92) (Extended Data Fig. 4d–f) and that pseudobulk profiles recapitulated information from RNA-seq (*r* = 0.85) (Extended Data Fig. 4g). Critically, data from these experiments also revealed that M3-seq libraries require ~15-fold fewer reads per cell to detect the same number of genes as undepleted libraries (Fig. 1f). M3-seq thus provides biologically meaningful, rRNA-depleted transcriptomes at single-cell resolution.

### M3-seq reveals an acid-tolerant *E. coli* subpopulation

**Extended Data Fig. 5:**
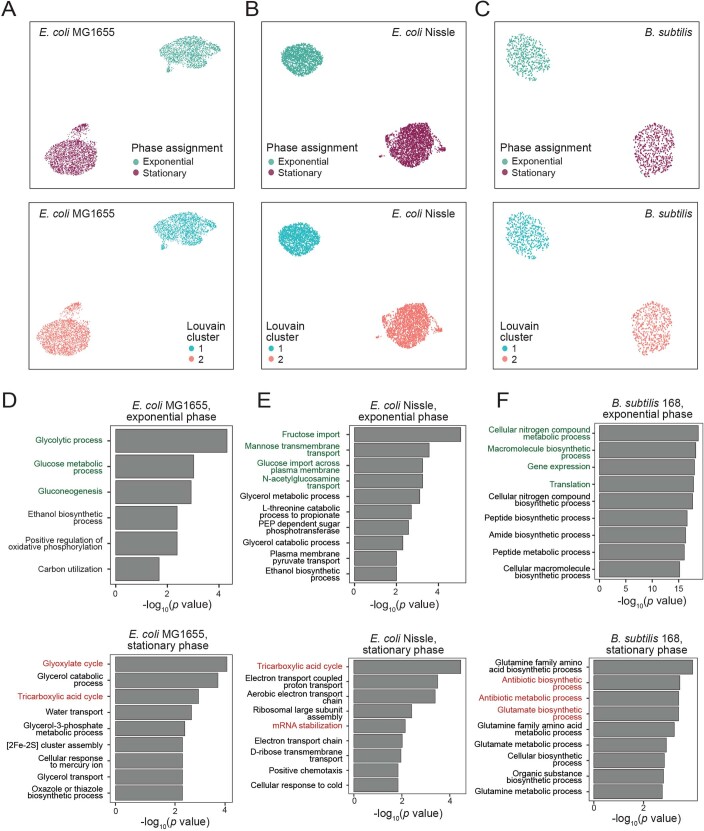
M3-seq profiling during exponential growth and early stationary phase. **a**. UMAPs of *E. coli* MG1655 transcriptomes in exponential and early stationary phase (top) and associated clustering (bottom, set to the lowest clustering resolution parameter). Clustering set at the lowest resolution parameter. Axes denote the first two UMAP components. **b**. Same as (A) but for *E. coli* Nissle. **c**. Same as (A) but for *B. subtilis* 168. **d**. GO term enrichment of select biological process calculated with marker genes identified for populations of exponential and stationary phase *E. coli* MG1655 identified in (A). Marker genes were determined as described in *Materials and Methods*. The *p*-values are -log_10_ transformed such that the most strongly enriched biological processes have the highest score. Selected processes were those with the lowest *p*-values after thresholding at 0.05. Enrichments for exponential and stationary phase cells include expected processes (green and red, respectively), including growth related and energy generation processes (exponential) and those involving secondary carbon metabolism and the TCA cycle (stationary). **e**. Same as (D) but for *E. coli* Nissle. Similar to *E. coli* MG1655, enrichments include expected processes (green for exponential; red for stationary). **f**. Same as (D) but for *B. subtilis* 168. Similar to *E. coli*, enrichments include expected processes (green for exponential; red for stationary).

The transition from exponential phase to early stationary phase represents a shift from rapid growth to slow growth as nutrients are depleted from the environment. Across the three bacterial strains in our eBW3 experiment, the transcriptomes from our single-cell data successfully distinguished stationary phase cells from those growing exponentially; that is, labelling groups of cells obtained with unsupervised clustering separated growth-stage-specific ‘round-one’ indices (Extended Data Fig. 5a–c). Gene ontology (GO) analysis of genes differentially expressed between those cells also showed clear enrichment for biological processes associated with one growth stage or the other (Extended Data Fig. 5d–f). As would be expected from dampened transcriptional output during slowed growth, stationary phase cells had substantially fewer UMIs per cell than did exponential-phase cells, with a median of 30 UMIs per cell for *B. subtilis* and *E. coli* MG1655 and 39 UMIs per cell for Nissle.

**Fig. 2:**
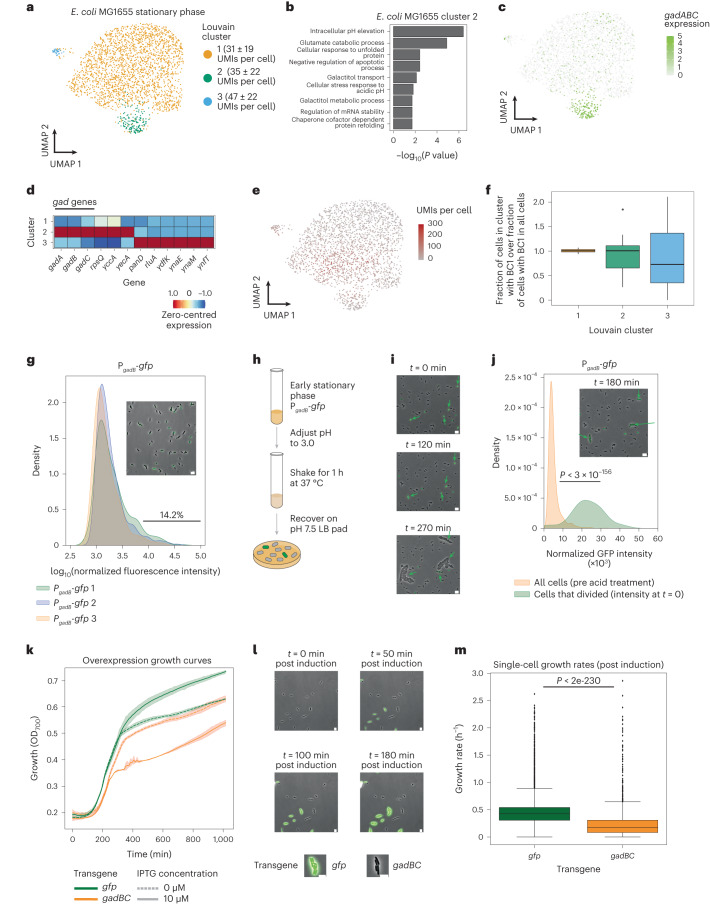
M3-seq reveals an acid-tolerant, bet-hedging subpopulation of *E. coli* in early stationary phase. **a**, Uniform Manifold Approximation and Projection (UMAP) of *E. coli* MG1655 transcriptomes from cells at early stationary phase (OD = 2.8). Colours indicate clusters of transcriptionally similar cells. **b**, GO-term enrichment of select biological processes calculated with marker genes identified for cluster 2 in **a**. Marker gene identification and GO-term analyses were performed as described in Methods. **c**, Same as **a** but with colour gradient indicating expression of *gadABC* genes (in normalized UMI counts). **d**, Zero-centred and normalized expression of marker genes for each cluster identified in **a**. Marker genes were determined as described in Methods. **e**, Same as **a** but with colour gradient indicating number of UMIs captured in each cell. **f**, Boxplot of normalized cluster percentage for each BC1 barcode in each cluster. The normalized cluster percentage and boxplot limits were determined as described in Methods. **g**, Normalized fluorescence distribution of early stationary phase *E. coli* transformed with P_*gadB*_*-gfp*. Inset is a representative composite image with phase and GFP channels overlaid. The *gad+* percentage was determined as described in Methods (*N* = *3* biological replicates, 5,034, 1,219 and 2,171 cells analysed, respectively*)*. Scale bar, 5 μm. **h**, Schematic of acid-stress recovery assay. Tubes are adapted from BioRender.com. **i**, Representative composite images of *E. coli* expressing P_*gadB*_*-gfp* during recovery phase of acid-stress recovery assay depicted in **h**. Arrows indicate cells that divided during the recovery period. Scale bar, 5 μm. **j**, Distributions of fluorescence intensity of *E. coli* expressing P_*gadB*_*-gfp* before and after acid-stress recovery assay. Orange depicts measurements from cells before acid treatment (1,833 cells) and green depicts measurements from cells a*t t* = 0 that ultimately divided over the course of recovery (that is, survived acid treatment, *N* = 38 cells). Inset is a representative composite overlay of the cells 180 min after the start of recovery from the same experiment as in **i**. Arrows indicate cells that divided during the recovery period. Scale bar, 5 μm. *P* = 2.99 × 10^−156^ from independent, two-sided *t*-test. **k**, Growth curves of *E. coli* MG1655 transformed with *gfp* or *gadBC* transgene (overexpression plasmids) and grown with or without 10 μM of IPTG (dashed curves) for 1,000 min. Curves indicate mean values and the shaded regions the 95% confidence interval of 3 technical replicates. **l**, Representative composite images of a mixed population of *E. coli* MG1655 transformed with *gfp* or *gadBC* transgene grown on an LB-agarose pad with 100 μM of IPTG. **m**, Single-cell growth rates of *E. coli* MG1655 transformed with *gfp* or *gadBC* transgene after transgene induction. Growth rates were computed as described in Methods from 539 and 112 observations, respectively, within a single set of videos (*N* = 1). *P* = 1.06 × 10^−230^ from independent, two-sided *t*-test. Boxplot limits are as defined in Methods.

**Extended Data Fig. 6:**
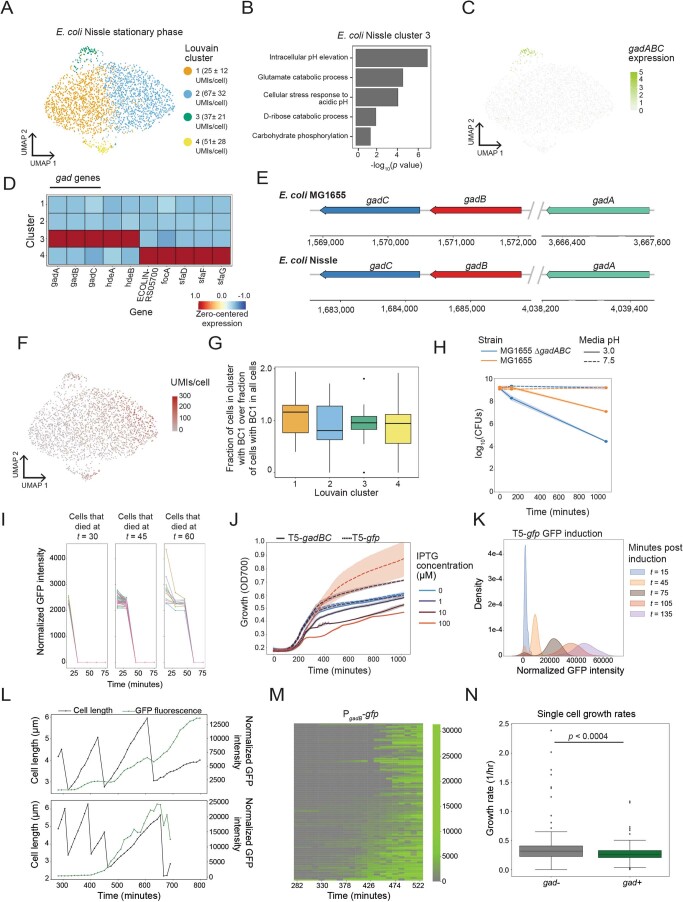
Subpopulation of early stationary phase cells expressing acid-tolerance genes also identified in *E. coli* Nissle. **a**. UMAP of *E. coli* Nissle transcriptomes from cells at early stationary phase (OD = 2.6). Colours indicate clusters of transcriptionally similar cells. **b**. GO-term enrichment of select biological processes calculated with marker genes identified for cluster 3 in (A). Marker gene identification and GO term analyses were performed as described in *Materials and Methods*. **c**. Same as (A) but with colour gradient indicating expression of *gadABC* genes (in normalized UMI counts). **d**. Zero-centred and normalized expression of marker genes for each cluster identified in (A). Marker genes were defined as described in *Materials and Methods*. **e**. Schematics of *gadABC* genes in the two strains of *E. coli* used in this study: MG1655 and Nissle. **f**. Same as (A) but with colour gradient indicating number of UMIs captured in each cell. **g**. Normalized cluster percentage for each BC1 in each cluster (*N* = 1, 1295, 1053, 83, 68 cells respectively). The normalized percentage for each BC1/cluster combination and boxplot limits are determined as described in *Materials and Methods*. **h**. Plot depicts survival of wildtype *E. coli* MG1655 and ∆*gadABC* mutant with and without exposure to acid stress during early stationary phase. Curves indicate mean values, and the shaded regions the 95% confidence interval between 2 biological replicates for control samples, and 4 biological replicates for acidified samples. **i**. Plot depicts fluorescence intensity of individual P_*gadB*_-GFP transformed *E. coli* MG1655 cells during acid exposure as described in *Materials and Methods*. Fluorescence intensity tracks are broken out by the time of death of each cell. **j**. Plot depicts growth of *E. coli* transformed with *gadBC* (solid) or *gfp* (dashed) transgene under different concentrations of IPTG inducer. Curves indicate mean values, and the shaded regions the 95% confidence interval between 3 technical replicates for each sample. **k**. Single-cell fluorescence distributions of *E. coli* transformed with GFP transgene after induction. **l**. Representative growth and GFP fluorescence intensity traces of *E. coli* transformed with P_*gadB*_-*gfp* during growth into stationary phase. **m**. Fluorescence kymograph of *E. coli* transformed with P_*gadB*_-*gfp* over time from (K). **n**. Single-cell growth rates of *gad-* and *gad+* cells from (L,M) using time-lapse microscopy. *gad-* and *gad+* cells were determined as described in *Materials and Methods* (*N* = 1, 93, 78 cells respectively). Growth rates were computed as described in *Materials and Methods*. *p* = 0.00032 obtained from independent, two-sided *t*-test.

In addition to differences between cells collected at different growth stages, we observed striking transcriptional heterogeneity ‘within’ populations of *E. coli* in early stationary phase cells (Fig. 2a and Extended Data Fig. 6a). A closer examination of cells from this growth stage revealed a cluster of cells overexpressing genes involved in intracellular pH elevation and glutamate catabolism (Fig. 2b and Extended Data Fig. 6b). The most strongly expressed genes in these clusters were *gadA* and *gadB* (Fig. 2c,d and Extended Data Fig. 6c,d). These genes are well conserved among enteric bacteria and are known to encode glutamate decarboxylases that de-acidify the cellular cytoplasm by consuming a proton during decarboxylation of glutamate to GABA (γ-aminobutyric acid) (Extended Data Fig. 6e)^20–22^. While previous studies have shown that these genes are expressed in stationary-phase *E. coli* using bulk measurements^23,24^ and heterogeneous expression has been observed in other conditions^25,26^, heterogeneous expression of *gadA* and *gadB* during the transition into stationary phase has not been previously reported. Before exploring these subpopulations further, we confirmed that total UMIs per cell for these particular subpopulations were not obviously different from the whole population (Fig. 2a,e and Extended Data Fig. 6a,f) and that neither cluster was substantially enriched for any particular round-one index (Fig. 2f and Extended Data Fig. 6g), which could indicate a technical artefact. We then moved on to experimental validation. Transforming *E. coli* MG1655 with a plasmid encoding a GFP variant (GFPmut2) controlled by the *gadB* promoter (P_*gadB*_-*gfp*) and imaging after growth in the same condition used for single-cell sequencing (Fig. 2g, inset) revealed 14.2% of cells expressing high levels of GFP controlled by the *gadB* promoter, which is comparable to 9.8% of cells from M3-seq experiments in early stationary phase *E. coli* with at least one transcript of *gadA* or *gadB* (Fig. 2g).

Our finding that *gad* genes are heterogeneously expressed in early stationary phase presented an opportunity to investigate the function of heterogeneous gene expression during a biologically important process. We first confirmed a functional role for the *gad* genes in our cells by asking whether *E. coli* MG1655 lacking *gadABC* can survive acid stress applied during early stationary phase (Extended Data Fig. 6h). Data from this experiment, which measured the number of viable cells by counting colony-forming units (c.f.u.s) with and without acid stress revealed that acid tolerance in the triple deletion strain was strongly impaired relative to wildtype. However, given the experimental design, these data could not link surviving cells to any pre-existing subpopulation. We therefore next deployed our P_*gadB*_-*gfp* reporter strain to monitor how cells with varying levels of *gadB* expression recover from acid treatment (Fig. 2h,i). First, we grew the reporter strain to early stationary phase and, using imaging, confirmed that a subpopulation of the cells expressed GFP. Next, we exposed the whole population of cells to acid stress (pH 3.0) and after 1 h, transferred an aliquot of the stressed cells to a fresh LB-agarose pad (*t* = 0). We then imaged the cells for 8 h. Quantification of GFP intensity as a proxy for *gadB* expression across individual cells in pre- and post-treatment images revealed that the population of viable cells, which were those that could not be stained by propidium iodide and divided at least once during the recovery period, were those expressing high levels of GFP at the beginning of the recovery (Fig. 2j). This observation suggests that the subpopulation of cells expressing high levels of *gadB*-driven GFP before acid exposure are the ones that subsequently survived acid treatment. Further supporting this possibility, imaging of early stationary P_*gadB*_-*gfp* reporter cells during strong acid stress found that rather than increasing in response to acid treatment, GFP fluorescence intensity steadily decreased in bacterial cells, probably due to reporter denaturation; however, cells that had high levels of GFP fluorescence at the beginning survived longer (Extended Data Fig. 6i and Supplementary Video 2). Together, these observations suggest that under sudden strong acid stress, early stationary phase *E. coli* do not induce a new *gad+* subpopulation to tolerate acid stress, but instead tolerate stress by relying on an existing subpopulation of *gad+* cells.

A reason for having only a subpopulation of cells expressing the *gad* genes during early stationary phase would be if there is a cost to expressing these genes. Using an overexpression system^27,28^, we observed a reduction in final cell density at the bulk level and a growth defect at the single-cell level (*P* < 2 × 10^−230^, Fig. 2k–m and Extended Data Fig. 6j,k). Furthermore, time-lapse microscopy of P_*gadB*_-*gfp* reporter cells during entry to stationary phase revealed asynchronous activation of *gadB*-driven GFP (Extended Data Fig. 6l,m) and a growth defect of GFP-high cells (gad+ cells) compared with GFP-low cells (*gad−* cells, *P* < 0.0004, Extended Data Fig. 6n). Paired with our functional characterization of the *gadB*-expressing subpopulation, these data suggest a model wherein *E. coli* can preemptively activate the *gad* genes to protect against future strong acid stresses (for example, such as would be experienced when passing through acidic environments such as the stomach), but because *gad* expression causes decreased growth overall, activation is limited to a subpopulation in case the acid stress does not materialize.

### Bacteriostatic antibiotics cause transcriptional variability

**Fig. 3:**
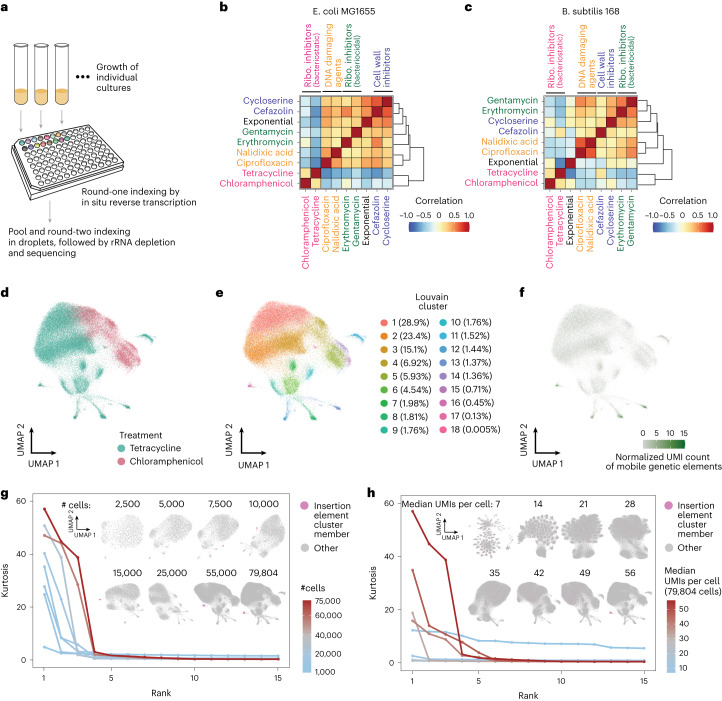
M3-seq enables systematic investigation of bacterial response to antibiotic treatment. **a**, Schematics of antibiotic experiment (eBW4). During preparation of M3-seq gene expression libraries, round-one plate indexing was used to uniquely mark antibiotic-treated and untreated cultures. Plate and tubes are adapted from BioRender.com. **b**, Heat map depicts Pearson correlations of pseudobulk transcriptomes from *E. coli* MG1655 prepared as in **a**, which were computed using genes with average expression greater than the median average expression of all genes. Coloured text indicates antibiotics of similar mechanisms of action. **c**, Same as **b** but for *B. subtilis* 168. **d**, UMAP of *E. coli* MG1655 transcriptomes from cells treated with the bacteriostatic antibiotics tetracycline and chloramphenicol. Colours indicate drug treatment. **e**, Same as **d** but with colours indicating clusters of transcriptionally similar cells. Percentage of cells in each cluster is denoted. **f**, Same as **d** but with colour gradient indicating the normalized UMI count of MGEs. Clusters 8, 12, 13 and 16 were enriched for MGE expression. **g**, Cell rarefaction analyses using M3-seq data. Curves indicate kurtosis of 15 principal components computed from tetracycline- and chloramphenicol-treated *E. coli* MG1655 cells, with individual curves corresponding to calculations from the total population of cells (79,804) or downsampled populations thereof (down to 1,000 cells). The 15 principal components included were those with the highest kurtosis for each downsampling. Inset UMAPs were computed from each downsampled data matrix. Within the embeddings, magenta indicates members of cluster 16 (indicated in **f**), which can only be distinguished for >7,500–10,000 cells. Notably, the top row of embeddings (2,500–10,000 cells) represents the scale of experiments from previous studies, while the bottom row represents the scale enabled by M3-seq. **h**, UMI rarefaction experiments using M3-seq data. Curves indicate kurtosis of 15 principal components computed from 79,804 tetracycline- and chloramphenicol-treated *E. coli* MG1655 cells, with individual curves corresponding to data subsampled for UMIs per cell (7 to 56 median UMIs). The 15 principal components included were those with the highest kurtosis for each subsampling of UMIs. Inset UMAPs were computed from each subsampled data matrix. Within the embeddings, magenta indicates members of cluster 16 (indicated in **f**), which can only be distinguished at the highest UMI detection efficiency.

**Extended Data Fig. 7:**
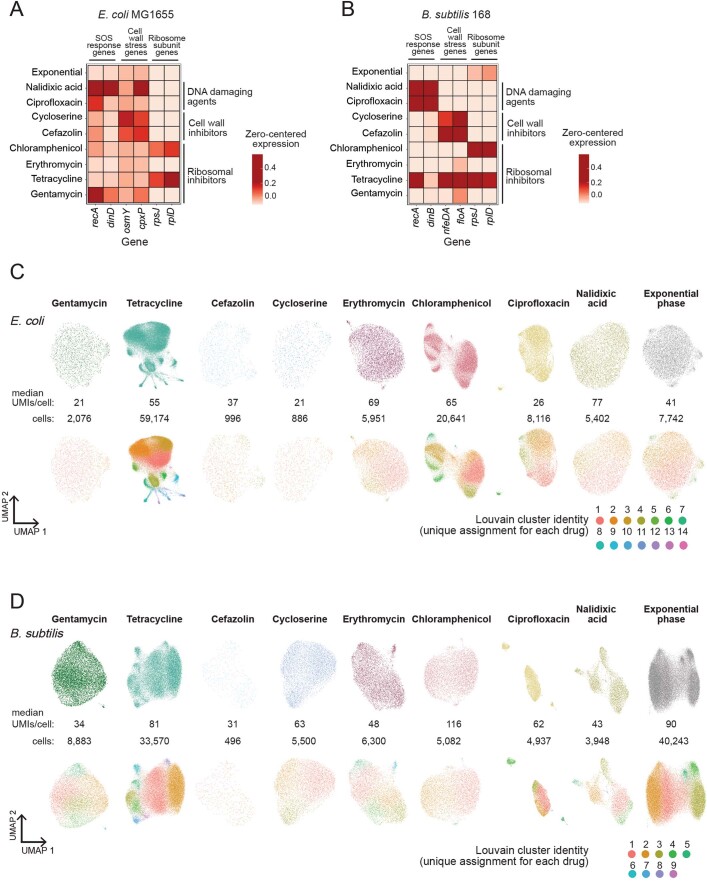
Multiplexed single-cell analysis of bacterial response to eight different antibiotics. **a**. Zero-centered and normalized expression of select genes in *E. coli* MG1655 cultures treated with the indicated antibiotics. Data from eBW4 (Supplementary Table 2). Genes were selected from among those related to the following GO terms: ‘Response to DNA damage’, ‘Cell wall stress’, and ‘Ribosome’. **b**. Same as (A) but for *B. subtilis*. Genes were selected from among those related to the ‘Response to DNA damage’ and ‘Ribosome’ GO terms and by searching for genes known to be upregulated in response to treatment with cell-wall targeting antibiotics (that is cefuroxime). **c**. UMAPs of *E. coli* MG1655 transcriptomes after treatment with indicated antibiotics (top) and corresponding cluster assignments (bottom). Clusters were uniquely defined for each population. **d**. Same as (C) but for *B. subtilis* 168.

How bacteria respond to antibiotic treatment is an important question. However, the large number of bacterial species and types of antibiotics, combined with variability of response within populations, makes this a difficult question to approach systematically. Combinatorial indexing provides a straightforward way to evaluate gene expression across many samples (that is, separate round-one indices can mark many cultures) and given the single-cell resolution of our platform, we reasoned that M3-seq could prove beneficial in this space. We therefore deployed M3-seq to evaluate bacterial cultures treated with each of eight antibiotics: two DNA-damaging agents (nalidixic acid, ciprofloxacin), two inhibitors of cell wall synthesis (cycloserine, cefazolin) and four ribosomal inhibitors (chloramphenicol, erythromycin, tetracycline, gentamycin) (Fig. 3a, and Supplementary Tables 1 and 3). In this experiment (eBW4), cultures were grown to early exponential phase (OD = 0.3), treated with 2× the minimum inhibitory concentration of each drug for 90 min and subjected to M3-seq across 2 lanes of a Single Cell ATAC chip. Altogether, we report data for 20 conditions across 229,671 cells (Supplementary Table 2) from which we make two systems-level observations: (1) indicative of successful profiling, select genes with known associations to antibiotic-induced stresses had higher expression in expected cultures (Extended Data Fig. 7a,b) and (2) hierarchical clustering of correlations between pseudobulk expression profiles grouped drugs with the same mechanism of action. These results suggest that M3-seq is a promising tool for systematic analysis (Fig. 3b,c).

**Extended Data Fig. 8:**
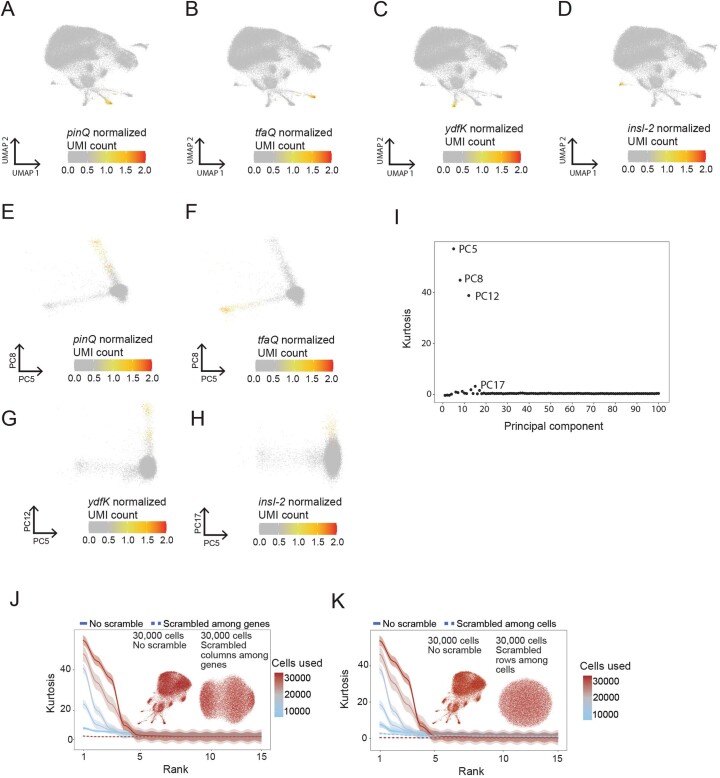
Defining MGE-expressing populations of *E. coli* using M3-seq data. **a**. UMAP of *E. coli* MG1655 transcriptomes from cells treated with the bacteriostatic antibiotics tetracycline and chloramphenicol. Colour gradient indicates normalized expression of *pinQ*, a marker gene for cluster 8 identified in Fig. 3e. **b**. Same as (A) but with colour gradient indicating normalized expression of *tfaQ*, a marker gene for cluster 13 identified in Fig. 3e. **c**. Same as (A) but with colour gradient indicating normalized expression of *ydfK*, a marker gene for cluster 12 identified in Fig. 3e. **d**. Same as (A) but with colour gradient indicating normalized expression of *insI-2*, a marker gene for cluster 16 identified in Fig. 3e. **e**. Plots of cells in principal component space for *E. coli* treated with bacteriostatic antibiotics, wherein the colour gradient indicates normalized *pinQ* expression. The principal component dimensions chosen for this analysis contained high loadings in genes that were upregulated in rare subpopulations (for example, *pinQ*, *tfaQ*). **f**. Same as (E) but with colour gradient indicating normalized *tfaQ* expression. **g**. Same as (E) but with colour gradient indicating normalized *ydfK* expression. **h**. Same as (E) but with colour gradient indicating normalized *insI-2* expression. i. Kurtosis of all 100 computed principal components calculated from the single-cell transcriptomes of tetracycline- and chloramphenicol-treated *E. coli* MG1655. Notably, principal components with the highest kurtosis were not necessarily the same as those with the highest variance. **j**. Kurtosis of 15 principal components computed from tetracycline- and chloramphenicol-treated *E. coli* MG1655 cells, with individual curves corresponding to calculations from down-sampled subsets of cells with and without UMI counts scrambled among genes. Notably, scrambling abolishes the kurtosis signal and removes structure from clustering. Curves indicate mean values, and the shaded region the 95% confidence interval across *N* = 5 independent down-samplings. **k**. Same as (J) but for down-sampled subsets of cells with and without UMI counts scrambled among cells across *N* = 5 down-samplings.

A closer examination of individual samples at the single-cell level (Extended Data Fig. 7c,d) revealed that tetracycline- and chloramphenicol-treated *E. coli* had a large number of transcriptional states (14 and 8 clusters, respectively) (Extended Data Fig. 7c and Supplementary Table 4). Unlike bactericidal drugs, such bacteriostatic agents do not have readily measurable single-cell persistence and tolerance phenotypes^3,29–31^, hence relatively little is known about heterogeneity in response to these drugs. Exploring the combined data from these two conditions identified several rare clusters that contained cells from both samples and expressed genes encoding mobile genetic elements (MGEs) (Fig. 3d–f, Extended Data Fig. 8a–d and Supplementary Table 4). Such rare cell populations may help cultures tolerate and escape the bacteriostatic state through subtle mechanisms (for example, activating genes implicated in cold shock, such as *ydfK*). From a technical perspective, these samples provided the largest number of transcriptomes from our experiment and high median UMIs per cell (Extended Data Fig. 7c)^32^. This high sampling undoubtedly enabled sensitive detection of rare populations but made direct comparison to other conditions difficult. Nevertheless, the large number of cells (79,804 from the two conditions combined) and high median UMIs (55 and 65 for tetracycline- and chloramphenicol-treated samples, respectively) within these populations provided an opportunity to evaluate requirements of scale and mRNA capture.

To better understand how the ability to detect rare subpopulations increases with the number of cells sequenced and UMIs captured, we first needed a metric capable of capturing transcriptional variability in the data. We found in our data that certain principal components had ‘heavy tails’, that is, outliers that strongly deviated from the mean loading for that principal component. These outlier cells were assigned as members of unique subpopulations in our clustering analysis (Extended Data Fig. 8e–h). We therefore reasoned that we could assess detection of rare cell subpopulations by computing the kurtosis (a measure of how heavy the tails of a distribution are) for each principal component (Extended Data Fig. 8i–k)^33^. Performing this analysis on random subsets of the data showed that the kurtosis of the top principal components (ranked by kurtosis) decreased when the data were downsampled (Fig. 3g,h). Correspondingly, a cluster containing the rare cell populations expressing *insI-2* was undetectable when clustering (Louvian with default parameters) downsampled data, with no ability to detect at lower cell numbers and UMI capture rates, including those relevant to other samples from this experiment, as well as previous studies (~1,000–5,000 cells, 7–49 UMIs per cell). This population nevertheless became apparent above our downsampling of 7,500 cells and 56 UMIs per cell. Notably, the kurtosis of the ‘heaviest’-tailed principal components monotonically increased with increasing cell numbers up to the number of cells in our experiment (79,804 cells) and the number of median mRNA transcripts captured (56 UMIs), suggesting that sequencing even more cells with deeper mRNA coverage could potentially identify even rarer subpopulations. Our combined analysis thus illustrates the need for scRNA-seq analysis to be performed at massive scale in bacteria and shows how M3-seq can enable such efforts.

### DNA-damaging antibiotics induce prophages in *B. subtilis*

**Fig. 4:**
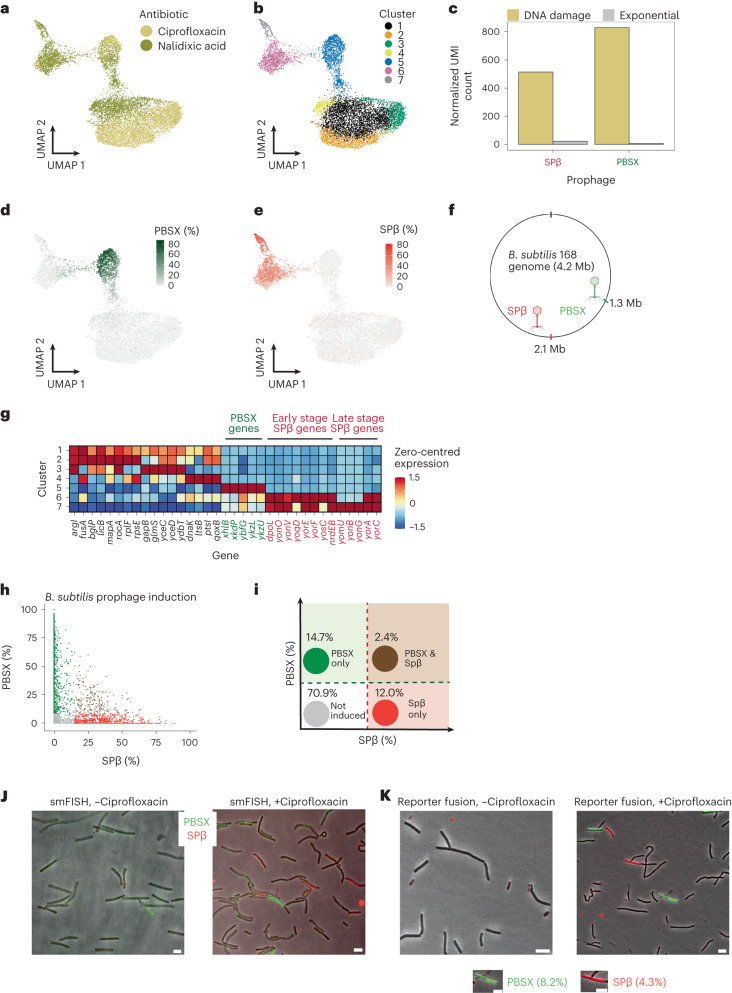
M3-seq characterizes independent activation of prophages in *B. subtilis*. **a**, UMAP of *B. subtilis* transcriptomes from ciprofloxacin- and nalidixic acid-treated cells in exponential phase (OD = 0.3). Colours indicate treatment conditions (90 min). **b**, Same as **a** but with colours indicating clusters of transcriptionally similar cells. **c**, Pseudobulk gene expression of the two prophages in the DNA-damaging antibiotic-treated conditions (yellow) compared to exponential phase (grey). **d**, Same as **a** but with colour gradient indicating percentage of PBSX prophage UMIs within each cell. Percentages were calculated by dividing the total number of PBSX UMIs by the total number of UMIs in each cell. **e**, Same as **a** but with colour gradient indicating percentage of SPβ prophage UMIs within each cell. **f**, Schematic of *B. subtilis* genome with location of PBSX and SPβ prophages indicated. **g**, Zero-centred and normalized expression of marker genes for each of 7 clusters identified in **b**. Marker genes were defined as detailed in Methods, where a maximum of 5 genes were included per cluster. **h**, Classification of cells with induced prophages. Green indicates cells with relative expression of PBSX genes >8.4% per cell, which is >10th percentile of PBSX prophage gene expression in cluster 5 from **b**. Red indicates cells with relative expression of SPβ genes >15.0% per cell, which is >10th percentile of SPβ prophage gene expression in cluster 6 from **b**. Brown indicates cells above both thresholds. **i**, Schematic of prophage classification results. The expected independent co-induction probability (calculated from observed PBSX and SPβ percentages) is 2.5%. **j**, Dual-colour smFISH of *B. subtilis* with no-drug treatment (left), or *B. subtilis* treated with ciprofloxacin for 90 min (right). Probes hybridizing to PBSX genes were labelled with a green fluorophore. Probes hybridizing to SPβ genes were labelled with a red fluorophore. Scale bar, 5 μm. **k**, Fluorescent reporter fusions of *B. subtilis* P_*L*_*-gfp* (PBSX promoter) and P_*yonO*_*-mKate2* (SPβ promoter) treated with no drug (left), or treated with ciprofloxacin (right) for 150 min to allow for maturation of the fluorescent protein. Percentages of induction were calculated from a single set of acquired images (*N* = 1,394 cells). Scale bar, 5 μm.

A second observation from our antibiotic study was that *B. subtilis* cells treated with DNA-damaging antibiotics (ciprofloxacin and nalidixic acid) exhibited a variety of transcriptional states (Fig. 4a,b and Extended Data Fig. 7d). Clustering the data and identifying marker genes associated with each cluster revealed that clusters 5, 6 and 7 had distinct sets of strongly co-expressed genes belonging to the PBSX or SPβ prophages (Fig. 4b–g). These prophages (PBSX and SPβ) are known to be induced by conditions that induce the SOS response such as DNA damage^34^, and both previous single-cell studies and our data have found that the PBSX prophage is induced in a small fraction of exponentially growing *B. subtilis*^10^, cluster 6 in exponential-phase *B. subtilis* (Extended Data Fig. 7d and Supplementary Table 4).

The heterogeneity of prophage induction we found in our single-cell data provided the opportunity to address an outstanding question: At the level of individual cells, is prophage induction stochastic or determined by some common perturbation (that is, degree of damage) or cross-talk (that is, co-repression)? Suggestive of stochastic induction, our analysis separated prophage-expressing cells into three groups: one dominated by PBSX-expressing cells (cluster 5) and two dominated by SPβ-expressing cells (clusters 6 and 7) (Fig. 4g and Supplementary Table 4). Further, on a per cell basis, comparison of PBSX and SPβ transcript percentages showed no obvious correlation (Fig. 4h) and rates of co-induction across cells, which we determined by thresholding, closely matched an assumption of independence (2.44% observed, 2.47% expected) (Fig. 4i). Therefore, we found no evidence for cross-repression or for a model wherein individual cells with the greatest damage had the greatest likelihood of inducing both prophages. Validation of prophage induction using single-molecule fluorescent in situ hybridization (smFISH) and using fluorescent reporter fusions on ciprofloxacin-treated cells, which we performed with probes against or reporter fusions for the most strongly expressed PBSX and SPβ genes, further supported this conclusion (8.2% cells inducing PBSX, 4.3% cells inducing SPβ) (Fig. 4j,k).

### Single-cell profiling of phage-infected bacteria

**Fig. 5:**
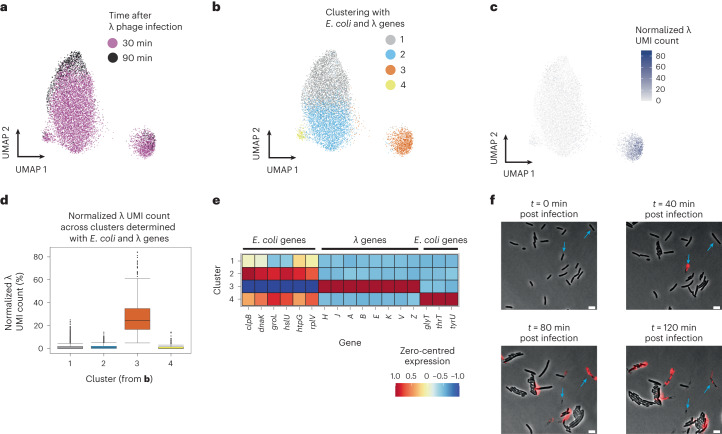
M3-seq reveals limited host response to heterogeneous λ phage infection. **a**, UMAP of λ phage-infected cells generated using alignments to both *E. coli* MG1655 and λ phage genomes (‘combined genome’). Colours indicate sampling timepoint after infection. **b**, Same as **a** but with colours indicating clusters of transcriptionally similar cells. **c**, Same as **a** but with colour gradient indicating normalized λ phage UMI count in each cell. Cluster 3 from **b** is strongly enriched for λ transcripts. We refer to this group of cells as the ‘lytic cluster’. **d**, Boxplots of normalized λ UMI count across each cluster in **b** (*n* = 4,326, 3,697, 1,189, 172). Boxplot limits are as defined in Methods. **e**, Zero-centred and normalized expression of marker genes for each of 4 clusters identified in **b**. Marker genes were identified as described in Methods. **f**, Representative composite time-lapse images of *E. coli* MG1655 infected with λ phage at MOI = 100. The red channel is a propidium iodide stain indicating cell death. Of cells in the initial frame, 34.1% were lysed. Data were collected from a single set of acquired images (*N* = 1,300 cells). Scale bar, 5 μm.

**Extended Data Fig. 9:**
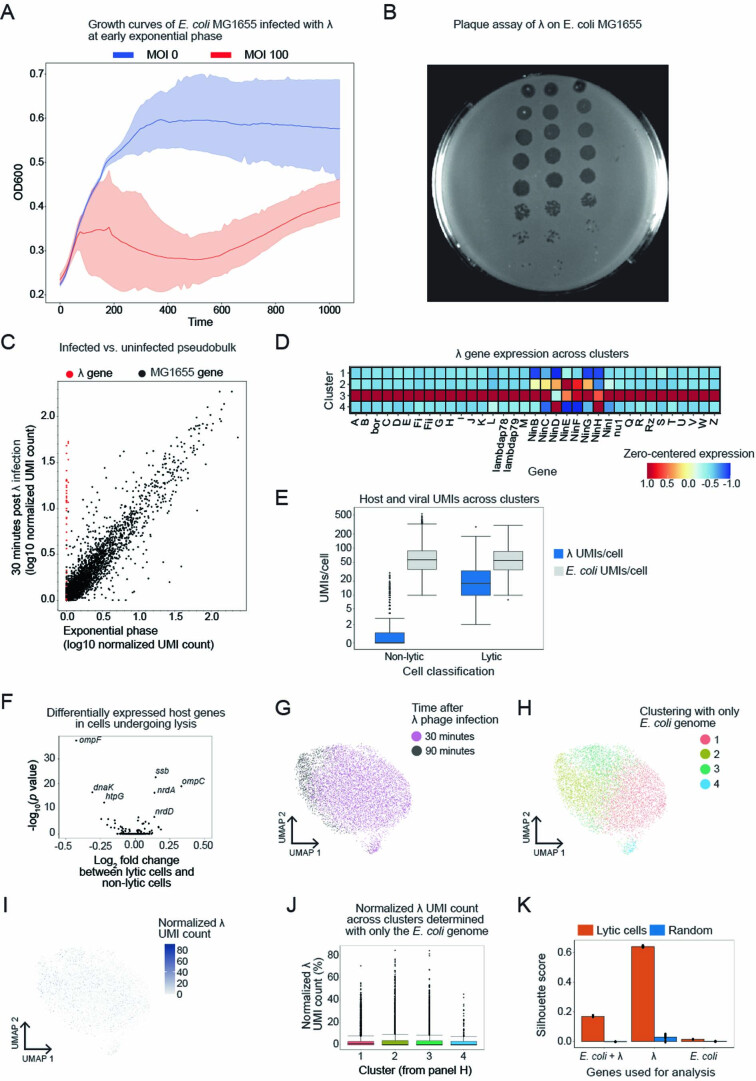
Growth and gene expression in *E. coli* cells infected with λ phage. **a**. Growth and gene expression in *E. coli* cells infected with λ phage. A. Plot depicts growth of *E. coli* grown to early exponential phase (OD ~ 0.2–0.3) and infected with λ phage (MOI ~ 100) or supplemented with phage vehicle (LB). Curves indicate mean values, and shaded error bars are 95% confidence intervals. **b**. Replicate plaque assays of λ phage grown on *E. coli* MG1655 without magnesium (the same conditions used in phage infection experiments eBW4). **c**. Pseudobulk comparison of the infected sample compared to an exponential phase control. Each point represents a single gene, and the red dots represent λ phage genes. **d**. Zero-centered and normalized expression of all observed λ genes for each cluster identified in Fig. 5b. Genes displayed were those genes which had more than 10 UMIs across the entire population. Expression of λ genes is strongly enriched in the lytic cluster (3) but lower in the rest of the population. **e**. Boxplot of *E. coli* and λ UMIs/cell of lytic (1189 cells) and non-lytic cells (8195 cells). Boxplot limits are as defined in *Materials and Methods*. We report a median of 57 ± 35 *E. coli* UMIs, 0 ± 0 λ UMIS for non-lytic cells, and 55 ± 34 *E. coli* UMIs, 18 ± 14 λ UMIs for lytic cells. Data was collected in a single sequencing experiment (*N* = 1). **f**. Volcano plot of all host genes when comparing the cells in the lytic cluster to cells outside the cluster. Fold changes and *p*-values were computed using the FindMarkers function in Seurat, where the ‘min.pct’ and ‘logfc.threshold’ were both set to 0. **g**. UMAP of phage infected cells generated using alignments to only the *E. coli* MG1655 genome. Colors indicate sampling timepoint after infection. **h**. Same as (G) but with colors indicating clusters of transcriptionally similar cells assigned after re-performing clustering with only *E. coli* transcripts. **i**. Same as (G) but with colour gradient indicating normalized λ phage UMI count in each cell. **j**. Boxplots of normalized λ UMI count across each cluster in (H) (*N* = 4215, 2885, 2075, 209 cells). Boxplot limits are as defined in *Materials and Methods*. **k**. Silhouette scores computed using the principal components of the lytic cluster (see Fig. 5b, c) and of ‘null subpopulation’ which is a random sample of cells across each alignment.

After observing gene expression from prophages, we reasoned that M3-seq could also be useful for studying active phage infection. Previous studies have evaluated transcriptional responses to phage with bulk measurements^35,36^, but variability of phage adsorption and infection from cell to cell limits interpretation of these data^37–39^; that is, bulk measurements can miss effects present only in rare populations or give the false impression that strong effects are homogeneous across a population. To address this limitation, we characterized gene expression in individual *E. coli* cells after infection with λ phage as part of eBW4. Briefly, we infected exponential phase *E. coli* MG1655 (grown to OD = 0.3) with λ phage at a multiplicity of infection (MOI) of ~100 (Extended Data Fig. 9a,b). We sampled the cultures at 30 and 90 min post infection, performed M3-seq and aligned the sequencing reads to a combined *E. coli* and λ genome. Comparing pseudobulk profiles from infected cells to those from exponential phase demonstrated an upregulation of λ genes, similar to previously reported data (Extended Data Fig. 9c)^35^. However, the single-cell transcriptomes formed four distinct clusters, with only one cluster (3) demonstrating high levels of λ gene expression (Fig. 5a–e).

During lysis, λ overtakes the host transcriptional machinery to express high levels of the late-stage genes required to produce functional virions. Indicative of lytic infection, cluster 3 revealed particularly high levels of late-stage λ genes (that is, *H, A, B, E, J, K*) (Fig. 5d, Extended Data Fig. 9d and Supplementary Table 4). By contrast, the most highly expressed genes in the remaining clusters (1, 2 and 4) were from *E. coli* and these non-lytic cells had similar levels of host UMIs as lytic cells (57 median UMIs for non-lytic cells, 55 median UMIs for lytic cells) (Fig. 5e, Extended Data Fig. 9e and Supplementary Table 4). Given the saturating MOI used in the experiment, these results were surprising. Our expectation was that all cells would be infected. To validate our measurements, we thus performed time-lapse microscopy on similarly infected *E. coli* cells and found that only 34.3% of cells in the initial frame eventually lysed, which agrees with the 33.6% of cells we observed by M3-seq to have >1 λ transcript at the 30-min timepoint (Fig. 5f). Collectively, these data show how even at high MOIs, bulk measurements do not accurately reflect the single-cell-level processes occurring during infection^40^.

Using our M3-seq data, we next sought to determine whether *E. coli* mount an active transcriptional response to λ infection and lysis. Examining host genes that were differentially expressed between the lytic cluster and the rest of the population revealed only a small set of genes with modest log_2_ fold changes (Extended Data Fig. 9f) and the upregulated genes encoded products previously reported to be part of indirect effects of lysis^35^. Reanalysing our data using only the *E. coli* MG1655 genome next revealed that without inclusion of the phage genes, cells identified with high viral load from analysis with the λ genome were not discriminated (Extended Data Fig. 9g–k)^40^. These results strengthen previous claims made using bulk transcriptional assays^35^ that *E. coli* do not mount a specific transcriptional response to λ phage lysis, despite the hijacking of host transcriptional machinery and the production of hundreds of foreign virions within the cell.

## Discussion

While emerging technologies for scRNA-seq provide a means to identify and characterize rare subpopulations of bacteria, many meaningful applications will require the ability to sequence large numbers of single cells across a diversity of experimental manipulations. Here we report the development of M3-seq, a two-step procedure of combinatorial indexing and efficient post hoc ribosomal RNA depletion that simultaneously enables scale in the number of cells profiled (herein, 229,671 total cells and 10,937 cells per condition), breadth in the number of conditions (herein, 20) and a high mRNA detection efficiency (herein, 100–1,000 UMIs per cell) (Fig. 1a). M3-seq therefore allows transcriptome-scale scRNA-seq at massive cell numbers and across multiple conditions. Alternative methods, including other combinatorial indexing-based approaches, can provide reasonable scale with comparable UMI capture, but most have an abundance of rRNA reads in the final library^9,10^. Established probe-based approaches have the opposite problem. By design, these methods avoid signal from rRNA but, due to the strain-specificity of probe hybridization, are not readily applied across species^12^. Moreover, techniques that rely on imaging may capture only up to a hundred genes at a time^11^. We note that concurrent with this study, two studies also reported using rRNA depletion in conjunction with bacterial scRNA-seq^15,41^. One of the described methods, BacDrop, uses an in situ enzymatic approach on unamplified transcripts before indexing and depletes rRNA to similar levels as we observed but risks digesting non-rRNA transcripts^15^. The other method^41^ uses a post hoc Cas9-based approach to deplete the amplified DNA library. This approach achieves less rRNA depletion^41^, which is consistent with our trial runs using Cas9-based rRNA depletion (75–80% rRNA in the final library, Extended Data Fig. 3a).

Despite the advantages of M3-seq, some technical challenges remain. One way to improve the method would be to develop a means of balancing the number of cells recovered across treatments; for example, we recovered ~59,000 tetracycline-treated *E. coli* cells in eBW4, but only 886 cycloserine-treated cells, which may represent a biological effect but is currently difficult to separate from possible technical considerations (for example, differences in round-one barcode capture). Looking forward, application of all current methods to mixed-species bacterial communities will also require computational solutions for parsing genes with highly conserved sequences and experimental optimization of in situ barcoding to maximize recovery of species-specific transcriptomes. Such challenges are highlighted in our study. For example, in one of our experiments (eBW4), we attempted to profile four species of bacteria (*B. subtilis, E. coli, Pseudomonas aeruginosa, Staphylococcus aureus*) but found that we could not recover UMIs at a satisfactory capture rate for the last two species. We attributed this challenge to growth stage differences, physical differences and sequencing depth. Nevertheless, the success of detecting multiple species and conditions in these experiments provides precedent for what we anticipate will be many applications of M3-seq to exploring niches and single-cell strategies that emerge within a microbial community.

We see multiple biological systems for which our technology is ripe to be applied. Undoubtedly, a key application will be host–pathogen interactions, for example, to reveal how bacteria mobilize phage-immunity mechanisms. Moreover, this application need not be restricted to bacterial cells. Because of the generality of using random primers and the rRNA depletion scheme, our method can also be employed to study how mammalian cells respond to infection by intracellular pathogens and how these infecting pathogens respond to host factors.

Why do rare bacterial subpopulations exist within a genetically identical bacterial population? One reason may be that transcriptional heterogeneity can act as a bet-hedging strategy in response to environmental variation. Such effects have been challenging to study with previous methods but using M3-seq, we discovered a rare acid-tolerant subpopulation expressing the *gad* genes in *E. coli*. We found that *gad*-expressing bacteria could survive strong acid treatment but were less fit in standard growth conditions, supporting a bet-hedging model of gene expression and highlighting how even temporally heterogeneous processes can have functional impact. Indicative of scRNA-seq as a discovery platform, many questions remain about this observation: How do varying environments change the presence of this subpopulation? How do other species such as *B. subtilis* deal with similar sorts of stresses? Similarly, through the scale afforded by M3-seq, we were able to uncover subpopulations of cells in *E. coli* exposed to bacteriostatic drugs, the biological relevance of which remains to be fully understood. Undoubtedly, additional bacterial single-cell profiling efforts will yield further understanding of these features in the future.

## Methods

### Bacterial strains and growth conditions for eBW1

*B. subtilis* 168 and *E. coli* (MG1655) were streaked out from a frozen glycerol stock onto an LB plate and grown overnight at 37 °C. Following a night of growth, a single colony was picked and inoculated into 5 ml of LB broth and grown with shaking at 250 r.p.m. overnight at 37 °C. The next morning, the overnight culture was diluted (1:100 for *E. coli*, 1:25 for *B. subtilis*) into multiple 30-ml tubes with 5 ml of fresh LB media and grown with shaking at 250 r.p.m. Cells were collected once at OD = 0.6 and again at 4 h post dilution. The volume of cells was normalized so that 1 OD of cells was sampled and fixed at each step. Cells were immediately spun down for 5 min at 5,000 *g* at 4 °C and resuspended in 4 ml of freshly made 4% formaldehyde. The resuspended cells were rotated overnight at 4 °C until the next morning.

### Bacterial strains and growth conditions for eBW2

*B. subtilis* 168 and *E. coli* (MG1655) were streaked out from a frozen glycerol stock onto an LB plate and grown overnight at 37 °C. Following a night of growth, a single colony was picked and inoculated into 5 ml of LB broth and grown with shaking at 250 r.p.m. overnight at 37 °C. The next morning, the overnight culture was diluted (1:100 for *E. coli*, 1:25 for *B. subtilis*) into 35 ml of fresh LB medium in a 250 ml Erlenmeyer flask and grown with shaking at 250 r.p.m. Upon reaching OD = 0.3, 5 ml of cells were split into tubes containing 2× the minimum inhibitory concentration of antibiotics (ciprofloxacin or cefazolin, 2 tubes), or no drug (2 tubes). The cells in the no-drug tubes were sampled once at OD = 0.6 and again at 120 min after the split. The cells in the tubes with drugs were sampled at 20 min post split (*T*_20_) and again at 120 min post split (*T*_360_). The volume of cells was normalized so that 1 OD of cells was sampled and fixed at each step. Cells were immediately spun down for 5 min at 5,000 *g* at 4 °C and resuspended in 4 ml of freshly made 4% formaldehyde. The resuspended cells were rotated overnight at 4 °C until the next morning.

### Bacterial strains and growth conditions for eBW3

*B. subtilis* 168 and *E. coli* (MG1655 and Nissle) were streaked out from a frozen glycerol stock onto an LB plate and grown overnight at 37 °C. Following a night of growth, a single colony was picked, inoculated into 5 ml of LB broth and grown with shaking at 250 r.p.m. overnight at 37 °C. The next morning, the overnight culture was diluted (1:100 for *E. coli*, 1:25 for *B. subtilis*) into 35 ml of fresh LB medium in a 250-ml Erlenmeyer flask and grown with shaking at 250 r.p.m. Upon reaching OD = 0.3, 5 ml of cells were split into tubes containing 2× the minimum inhibitory concentration of antibiotics (ciprofloxacin or cefazolin), or no drug. The cells in the no-drug tubes were sampled once at OD = 0.6 and again at 360 min after the split. The cells in the tubes with drugs were sampled at 90 min post split (*T*_90_) and again at 360 min post split (*T*_360_). The volume of cells was normalized so that 1 OD of cells was sampled and fixed at each step. Cells were immediately spun down for 5 min at 5,000 *g* at 4 °C and resuspended in 4 ml of freshly made 4% formaldehyde. The resuspended cells were rotated overnight at 4 °C until the next morning.

### Bacterial strains and growth conditions for eBW4

*B. subtilis* 168, *E. coli* MG1655 and *P. aeruginosa* PA14 were streaked out from a frozen glycerol stock onto an LB plate and grown overnight at 37 °C. Following a night of growth, a single colony was picked, inoculated into 5 ml of LB broth and grown with shaking at 250 r.p.m. overnight at 37 °C. The next morning, the overnight culture was diluted (1:100 for *E. coli*, 1:25 for *B. subtilis*, 1:50 for *P. aeruginosa*) into 35 ml of fresh LB medium in a 250-ml Erlenmeyer flask and grown with shaking at 250 r.p.m. Upon reaching OD = 0.3, 4 ml of cells were split into tubes containing 2× the minimum inhibitory concentration of antibiotics (gentamycin, tetracycline, erythromycin, chloramphenicol, cefazolin, cycloserine, ciprofloxacin, or nalidixic acid), λ phage at MOI = 100 (for *E. coli*), or no drug. The cells in the tubes were sampled and had their absorbance read at 90 min post split (*T*_90_). The volume of cells was normalized so that 1 OD of cells was sampled and fixed at each step. Cells were then prepared in the same manner as with eBW1–3.

### Cell preparation

Following an overnight fixation, cells were prepared for scRNA-seq following an adjusted protocol^9^. Briefly, cells were first spun down for 10 min at 5,000 *g* at 4 °C. Cells were then resuspended in 0.25 ml of PBS-RI comprising PBS + 0.01 U μl^−1^ SUPERase-IN RNase inhibitor (Invitrogen, AM2696). Cells were spun down again for 10 min at 5,000 *g* at 4 °C and resuspended in 150 μl of 1× PBS-RI and 150 μl of 100% ethanol. Following the first permeabilization, cells were spun down for 8 min at 7,000 *g* at 4 °C and washed twice with 200 μl of PBS-RI. After this final wash, cells were permeabilized by resuspension in 45 μl 2.5 mg ml^−1^ lysozyme solution dissolved in TEL-RI buffer comprising 100 mM Tris (pH 8.0), 50 mM EDTA and 0.1 U μl^−1^ SUPERase-IN RNase inhibitor and incubated at 37 °C for 15 min. Cells were then spun down and washed once in 100 μl of PBS-RI. After the final wash, cells were resuspended in 100 μl of 0.5× PBS-RI, counted and examined with a haemocytometer (INCYTO DHC-S02).

### Round-one indexing

Fixed and permeabilized cells were split into wells of a 96-well plate, each containing a single indexing primer (2.5 μl per well, 20 µM). To each well, we added 312,500 cells, 0.25 μl of Maxima H Minus reverse transcriptase (Thermo Fisher, EP0753), 0.25 μl of deoxyribonucleotide triphosphates (dNTPs) at an original concentration of 10 mM (NEB, N0447L), 2.5 μl of 5× Maxima H Minus reverse transcription buffer, 0.125 μl RNase-Out (Thermo Fisher, 10777019) and PEG 8000 to a final concentration of 7.5%, Tween-20 to a final concentration of 0.02% and nuclease-free water up to 10 μl. Reactions were then incubated as follows to perform first-round indexing by reverse transcription: 50 °C for 10 min, 8 °C for 12 s, 15 °C for 45 s, 20 °C for 45 s, 30 °C for 40 s, 42 °C for 6 min, 50 °C for 50 min and hold at 4 °C. Samples were then pooled together and spun for 20 min at 7,000 *g* to isolate processed cells. Cells were then washed in 0.5× PBS-RI and resuspended in 75 μl of 1× Ampligase buffer (Lucigen, A0102K). Pooled cells were counted and examined on the haemocytometer, and diluted for loading onto the Chromium Controller (10X Genomics). The cell loading for each experiment is indicated in Supplementary Table 2. Methods in this section were adapted from single-cell combinatorial fluidic indexing procedures.

### Loading cells into microfluidic droplets

Cells were prepared for loading onto the Chromium scATAC platform v.1.1 (10X Genomics 1000176). After counting, pooled cells were aliquoted and mixed with 19 μl 1× Ampligase buffer, 2.3 U μl^−1^ Ampligase (Lucigen A0102K), 1.5 μl reducing agent B (10X Genomics, 2000087), 2.3 μl 100 µM bridge oligo oDS025 and nuclease-free water up to 75 μl. The mixture was kept on ice and loaded onto the Chromium Next GEM Chip H (10X Genomics, 1000162) with gel beads from the Chromium Next GEM Single Cell ATAC Library & Gel Bead kit (10X Genomics, 1000176). To create emulsions, we followed the Chromium Single Cell ATAC Reagent Kits User Guide (v.1.1 Chemistry) (CG000209 Rev A). Briefly, the microfluidic chip was prepared by adding 70 μl of cell mixture to wells in row 1, 50 μl Next GEM scATAC beads to wells in row 2 and 40 μl of partitioning oil to wells in row 3. In addition, 50% glycerol was added to all unused lanes (70 μl 50% glycerol was added to unused lanes in row 1, 50 μl to unused lanes in row 2 and 40 μl to unused lanes in row 3). The chip was run on the Chromium Controller (10X Genomics) with the Next GEM Chip H programme. This step partitions the cells and uniquely indexed gel beads into droplets. Methods in this section were adapted from single-cell combinatorial fluidic indexing procedures^16^.

### Round-two indexing

After transferring 100 μl of each emulsion mixture to a clean reaction tube, second-round indexing was performed by ligation. Briefly, emulsions were incubated for 12 cycles of 98 °C for 30 s and 59 °C for 2 min. Emulsions were broken by adding 125 μl recovery agent (10X Genomics) and pipetting up the hydrophobic phase. Cells were then reverse crosslinked and lysed by adding 10 μl of 10× Lysis-T (250 mM EDTA, 2 M NaCl, 10% Triton X-100) and 4 μl of proteinase K (NEB, P8107S), and incubating at 55 °C for 1 h. After lysis, DNA:RNA hybrid libraries were isolated using the following procedure: (1) 200 μl of Dynabead cleanup mix, which consists of 182 μl cleanup buffer (10X Genomics, 2000088), 9 μl Dynabeads MyOne Silane (Thermo Fisher, 37002D), 5 reducing agent B (10X Genomics, no catalogue no.) and 5 μl of nuclease-free water, was added to each sample; (2) samples were mixed by pipetting (10×); (3) samples were incubated at room temperature for at least 10 min; (4) beads were isolated from samples using a magnetic stand and washed 2 times with 200 μl 80% ethanol; and (5) hybrid libraries were then eluted in 40 μl of elution buffer (Qiagen, 19086).

### Second-strand cDNA synthesis

The eluted single-stranded library was stripped of RNA by adding 2 μl of RNase H (NEB M0297L), 4 μl of 10× RNase H buffer (NEB B0297S) and incubating for 30 min at 37 °C. The reaction was purified with a 1.8× solid phase reversible immobilization (SPRI), where the final eluate volume was 25 μl. To perform second-strand synthesis, we used a modified version^42^, where we added 8 μl of 5× Maxima H- reverse transcription buffer, 4 μl 10 µM dNTPs, 2.5 μl of Klenow Fragment (3’ -> 5’ exo -, NEB M0212L), 5 μl 50% PEG 8000 and 1.5 μl 100 µM S^3 randomer (oBW140). The reaction was incubated at 37 °C for 60 min, cleaned with a 1.8× SPRI and eluted in 30 μl of nuclease-free water. The full length, double-stranded library was amplified using PCR by adding 30 μl of 2× Q5 High Fidelity master mix (NEB M0492L), 0.4 μl 100 µM oDS028 and 0.4 μl 100 µM oBW170. We amplified the library using the following protocol: 98 °C for 30 s, 14 cycles of 98 °C for 20 s, 65 °C for 30 s, 72 °C for 3 min. Following the first round of PCR, the reaction was cleaned twice, each time using a 1.2× SPRI reaction, and eluting in 40 μl. This was to ensure primer dimers were properly removed. The resulting samples were the gene expression (GEX) libraries.

### Library fragmentation using Tn5 transposase

We prepared the following 5× Tn5 reaction buffer: 50 mM N-[tris(hydroxymethyl)methyl]-3-aminopropanesulfonicacid (TAPS) (Sigma, T9659-100G), 25 mM MgCl_2_. We assembled Nextera Read 2-only transposomes according to established protocols^16^. Briefly, 10 μl 100 µM oDS029 and 10 μl 100 µM oDS30 were mixed and annealed using the following temperature programme: 95 °C for 2 min, followed by a 0.1 °C s^−1^ ramp down to 4 °C. Annealed oligos were then diluted with 80 μl of nuclease-free water (final concentration, 10 µM) and, after 10 μl 100% glycerol was added to an aliquot of 10 μl diluted annealed oligos, 8 μl of the oligo-glycerol sample was mixed with 2 μl of EZ-Tn5 (Lucigen, TNP92110) and incubated at 25 °C for 40 min. The resulting Read 2 transposomes were stored at −20 °C.

After construction, gene expression libraries were quantified (Qubit HS dsDNA kit) and fragmented in multiple reactions with the following components: 10 ng gene expression library sample, 4 μl of 5× Tn5 buffer, 1 μl of Read 2 transposome and water up to 20 μl. Reactions were incubated at 55 °C for 10 min and then inactivated with 1 μl 20% SDS at 55 °C for 10 min. Following inactivation, reactions were purified using a 1.2× SPRI reaction (elution volume, 25 μl). The resulting samples were the fragmented GEX libraries.

### Second library amplification and in vitro transcription

Fragmented GEX libraries were mixed with 25 μl of 2× Q5 master mix, 0.4 μl 100 µM oBW170 and 0.4 μl 100 µM oBW168, and amplified using the following protocol: 72 °C for 3 min, 98 °C for 30 s, 9 cycles of 98 °C for 10 s, 65 °C for 30 s, 72 °C for 30 s, a final incubation at 72 °C for 5 min and hold at 4 °C. Resulting samples were purified with a 1.2× SPRI reaction (elution volume, 40 μl) and converted into RNA by in vitro transcription. Briefly, 100 ng of amplified libraries were mixed with 8 μl 5× transcription buffer (Thermo Fisher, EP0112), 6 μl 2.5 mM rNTPs (NEB, N0466L), 1.5 μl of T7 RNA polymerase (Thermo Fisher, EP0112) and 1 μl of RNase-Out. Reactions were incubated at 37 °C for 2 h, after which DNA templates were digested with 3 μl DNase I (NEB, M0303L) and 3 μl 10× DNase I buffer (NEB, B0303S) at 37 °C for 15 min. RNA was purified using a 2× SPRI reaction (elution volume, 25 μl). These samples were the in vitro transcribed GEX libraries.

### Ribosomal RNA depletion

To enrich for mRNA reads within a DNA library that was constructed using random priming, we developed an in-house approach to deplete ribosomal reads. Probes hybridizing to ribosomal RNA sequences of the bacterial species used in this study were designed (using previously designed software^19^). Multiple reactions (depending on the yield of the in vitro transcription reaction) each containing 500 ng of RNA, probes, and hybridization buffer were prepared as follows (using protocols adapted from ref. ^19^): 500 ng of in vitro transcribed RNA, 3 µg of rRNA probes, 0.6 μl 5 M NaCl, 1.5 μl 1 M Tris-HCl and nuclease-free water up to 15 μl. Hybridization was then performed using the following temperature programme: 95 °C for 2 min and 0.1 °C s^−1^ ramp down to 25 °C, 25 °C for 5 min. Following rRNA probe hybridization, 6 μl RNase H mix consisting of 3 μl of 10× RNase H buffer (NEB B0297), 2 μl of thermostable RNase H (NEB M0523S) and 1 μl of RNase H were added to each tube. The reactions were incubated for 45 min at 50 °C to digest the rRNA–DNA hybrids. Following rRNA digestion, the DNA probes were degraded by adding 3 μl of 10× DNase I buffer, 3 μl of DNase I and incubating for 45 min at 37 °C. The rRNA-depleted RNA library was purified with a 2× SPRI reaction and eluted in 25 μl of nuclease-free water.

### Final library prep

To recover an rRNA-depleted cDNA library for sequencing, we next performed a second round of reverse transcription using the end specific P5 primer, thus ensuring reverse transcription of full library constructs. To each tube of purified RNA, we added the following reagents: 8 μl Maxima H Minus reverse transcription buffer, 1 μl Maxima H Minus reverse transcriptase, 1 μl RNase-Out, 6 μl 2.5 mM dNTPs, 0.4 μl 100 µM oBW170 and 0.2 μl 100 µM oBW171. The reaction was incubated in the thermocycler with the following temperature programme: 50 °C for 10 min, 8 °C for 12 s, 15 °C for 45 s, 20 °C for 45 s, 30 °C for 40 s, 42 °C for 6 min, 50 °C for 18 min and hold at 4 °C.

Following reverse transcription, the reaction was purified with a 1.2× SPRI and eluted in 25 μl of nuclease-free water. The reverse-transcribed DNA reactions were then indexed using a final indexing PCR to multiplex different libraries on the same sequencing run. For each reaction, 25 μl of reverse-transcribed DNA was mixed with 25 μl Q5 High Fidelity master mix, 0.4 μl 100 µM oBW170 and 0.4 μl 100 µM of a unique P7 index primer. The reactions were amplified with the following temperature programme: 98 °C for 30 s, 9 cycles of 98 °C for 10 s, 65 °C for 30 s, 72 °C for 30 s, a final incubation at 72 °C for 5 min and hold at 4 °C.

After two purifications with 0.8× SPRI, our final sequencing libraries were quality controlled on the Qubit and Bioanalyzer. We also checked the concentration and quality of each DNA library using qPCR (primers: oBW170/oBW176, oBW141/oBW176). We note that this final qPCR step is essential as it checks for the percentage of the reads that can be sequenced in each library. Typically, a ΔCT of 0–0.6 (oBW141/oBW176 - oBW170/oBW176) indicates a fully sequenceable library. Following the final qPCR, libraries were diluted to 5 nM and sequenced with the NovaSeq SP 100 cycle kit (Illumina 20028401) using the following read structure: 26 bp Read 1, 30 bp i5 index, 8 bp i7 index, 74 bp Read 2.

### FISH

To enable cost-effective detection of multiple different RNAs in the same cells, we closely followed established frameworks for single-molecule FISH^43,44^. Briefly, multiple primary probes hybridizing to an mRNA of interest were first designed. These probes contained a constant 20-nt flanking sequence that allows for hybridization of a fluorescent secondary probe. This allowed us to avoid the cost of ordering multiple fluorescent primary probes to tile our gene of interest.

Primary probes for FISH for RNA sequences of interest were designed using the same software used to design rRNA probes^19^. For each RNA transcript of interest, we designed at least 10 different probes hybridizing to different regions of that transcript. A 20-nt sequence was added to the 3’ end of each probe to allow for hybridization of the fluorescent readout probes. Primary probes for each gene were mixed at an equimolar ratio such that the final concentration of DNA molecules was 100 µM. Fluorescent readout probes were ordered following Supplementary Table 1 in ref. ^44^.

Cells in each condition of interest were grown, fixed and permeabilized as described above. After the permeabilization step, cells were washed and resuspended in 600 μl primary hybridization buffer (40% formamide (Thermo Fisher, 15515026), 2× SSC (Invitrogen AM9673)) and aliquoted into 1.5 ml tubes. Primary probe mix (1 μl, 100 µM) was added to each tube and hybridized overnight at 30 °C in the dark. The next morning, cells were spun down at 7,000 *g* for 8 min and resuspended in 200 μl wash buffer (30% formamide (Thermo Fisher, 15515026), 2× SSC (Invitrogen, AM9673)). Cells were spun down for 8 min at 7,000 *g*, resuspended again in 200 μl wash buffer and incubated in the dark at room temperature for 30 min. Cells were then spun down at 7,000 *g* for 8 min and resuspended in 100 μl secondary hybridization buffer (10% formamide, 2× SSC, 10% Ficoll PM-400 (Sigma-Aldrich F5415-25 ml)). Of each 100 µM readout probe, 0.5 μl was added to the tubes and incubated for 1 h at 34 °C. Following secondary hybridization, cells were spun down at 7,000 *g* and resuspended in wash buffer with 10 µg ml^−^1 DAPI (Thermo Fisher, D1306). Cells were incubated for 20 min at room temperature, spun down at 7,000 *g* and resuspended in 100 μl of 2× SSC.

Cells were imaged on 1% agarose pads made with filtered PBS on a Nikon TiE microscope with a Plan Apo ×100 objective, Hanamatsu ORCAFlash4.0 camera and NIS Elements imaging software v.5.21.00. Images were analysed using FIJI v.2.9.0.

### Acid tolerance assay

A 25 ml culture of *E. coli* (MG1655) or *E. coli* (MG1655 *ΔgadAΔgadBΔgadC)* was first grown to OD = 0.3 in a 125 ml flask with shaking at 250 r.p.m. at 37 °C. After reaching OD = 0.3, the cultures were split in aliquots of 5 ml to culture tubes and placed back onto the shaker to grow for another 6 h until OD = 2.8. Cultures were then acidified to pH 3.0 using 12 N HCl and returned to the shaker. A volume of 10 μl of the cultures was sampled at intermittent timepoints and serial diluted for c.f.u. counting.

### Acid recovery assay

A 25 ml culture of *E. coli* (MG1655) transformed with P_*gadB*_*-gfp* was first grown to OD = 0.3 in a 125 ml flask with shaking at 250 r.p.m. at 37 °C. After reaching OD = 0.3, the cultures were split in aliquots of 5 ml to culture tubes and placed back onto the shaker to grow for another 6 h until OD = 2.8. At this point, 1 μl of the culture was imaged on a 1% agarose pad made with LB medium to understand the distribution of GFP fluorescence in single cells. Cultures were then acidified to pH 3.0 using 12 N HCl and returned to the shaker. Following 1 h of acid stress, 1 μl of the acidified culture was transferred onto a fresh 1% LB-agarose pad at pH 7.5 at 37 °C to assess viability. *t* = 0 refers to the time when cells were placed onto the pad. Cells were imaged every 15 min to track and assess growth over time.

The resulting movies were analysed by first segmenting the cells using DeLTa^45^ v.2.0.0 and then using custom Python scripts to extract the fluorescence distribution and assess viability. A cell was considered viable if it underwent a single division during the 8-h imaging period.

### Quantification of the *gad* subpopulation

Cells were grown as described above. Following the split into 5 ml aliquots, cells were allowed to grow for 6 more hours until OD = 2.8 and imaged on a 1% agarose pad made with filtered PBS.

Following data acquisition, cells were segmented and tracked using DeLTa and then analysed with custom scripts. To determine the percentage of *gad*+ cells within each replicate, we first log transformed the length-normalized fluorescence intensity of each cell and then fit a normal distribution to the log-transformed intensities^46^. Cells with fluorescence intensity beyond the 99th percentile of the theoretical distribution were considered as *gad+*. The percentage of *gad+* cells was then calculated using the number of *gad+* cells determined above.

### Imaging *E. coli* under strong acid stress

Cells were grown as described above. After reaching OD = 2.8, cells were transferred to a fresh LB pad adjusted to pH 3.5 with 1 μl of propidium iodide. Following data acquisition, cells were segmented and analysed to identify any GFP fluorescence change over time.

### Single-cell growth analysis into stationary phase

Cells were grown as described above. Following the split into 5 ml aliquots, cells were grown for 2 more hours in a 125 ml flask with shaking at 250 r.p.m. at 37 °C. These cells were then diluted 5-fold in conditioned media, and then 1 μl of cells were imaged on a 1% agarose pad made with DPBS at 30 °C. To track single-cell growth and fluorescence, cells were imaged every 12 min over a period of 10 h.

Following data acquisition, cells were segmented and tracked using DeLTa and then analysed with custom scripts. Growth rates were calculated as the change in segmented cell length per hour normalized using the cell length. Fluorescence intensity in each cell was normalized by using the cell area. To classify *gad+* and *gad*− in the time-lapse data, we took the top quartile of cells of GFP expression as *gad*+ and the bottom quartile as *gad*−. Growth rates were calculated during a 30-min window at 420–450 min after the start of imaging and significance values were computed using independent two-sided *t*-test.

### Single-cell growth analysis under IPTG induction

Cells were grown by backdiluting (1:100) overnights of *E. coli* (MG1655) transformed with either T5-*gfp* or T5-*gadBC* into 25 ml of LB in a 125 ml flask, with shaking at 250 r.p.m. at 37 °C. In the mixed culture experiment, after cells reached an OD = 0.3, 500 μl of each culture were mixed in an Eppendorf tube. Isopropylthio-β-galactoside (IPTG) was then added to a final concentration of 100 μM. Of the mixed culture, 1 μl was added to a 1% agarose pad made with LB with 100 μM IPTG. Cells were imaged every 10 min at 37 °C over a period of 3 h.

Following data acquisition, cells were segmented and tracked using the DeLTa software as described above and then analysed with custom scripts. Growth rates and fluorescence intensity were calculated as described above. In the mixed culture experiment, cells were identified as containing T5-*gfp* if the fluorescence intensity of a cell was more 10,000 fluorescence units. Growth rates of the two populations and the associated significance values were computed as described above.

### Imaging phage lysis

An overnight culture of *E. coli* MG1655 was backdiluted 1:100 into 25 ml of LB in a 125 ml flask, with shaking at 250 r.p.m. at 37 °C. Following growth to OD = 0.3, 500 μl of these cells were mixed with λ phage to an MOI = 100. A volume of 1 μl of cells + phage was added to a 1% agarose pad made with LB + 1 μl of propidium iodide, and λ phage added to the same concentration as for the cells. Cells were imaged every 10 min at 37 °C over a period of 4 h. Following data acquisition, cells were manually counted and tracked to find the total number of lysed cells over the first 120 min.

### Bulk RNA-seq library preparation

*E. coli* (MG1655) was grown as described above to OD = 0.6. A volume of 2 ml of cells was spun down at 5,000 *g* for 10 min, resuspended in 45 μl 2.5 mg ml^−1^lysozyme solution (described above) and incubated at 37 °C for 15 min. RNA was purified using the Qiagen RNeasy Mini kit (Qiagen, 74104) where the final eluate volume was 30 μl. The RNA was reverse transcribed by adding 5 μl Maxima H Minus reverse transcription buffer, 0.5 μl Maxima H Minus reverse transcriptase, 0.5 μl RNase-Out, 4 μl 2.5 mM dNTPs and 0.4 μl 100 µM oBW121, and incubating using the following temperature programme: 50 °C for 10 min, 8 °C for 12 s, 15 °C for 45 s, 20 °C for 45 s, 30 °C for 40 s, 42 °C for 6 min, 50 °C for 50 min and hold at 4 °C.

Following reverse transcription, RNA was stripped from the reverse-transcribed DNA by adding 2 μl of RNase H and incubating the mixture at 37 °C for another 30 min. The library was purified using a 1.2× SPRI and eluted in 25 μl nuclease-free water. Second-strand synthesis, PCR and tagmentation were performed as described above. The first PCR was performed using primer pairs oBW154 and oDS28. Following tagmentation, the library was amplified for 8 cycles as described above using oBW154 and oBW168. This library was used to test for different rRNA depletion strategies.

### Cas9-based rRNA depletion

To test Cas9-based rRNA depletion, we first synthesized a pool of guide RNAs that cleave at different sites of the 5S, 16S and 23S ribosomal RNAs. DNA templates for the guide RNAs were designed by running previously written scripts^17^. The 5S, 16S and 23S rRNA sequences of the species of interest were combined into a fasta file and used as input for the software, which was run with default parameters.

The DNA templates were purchased as a pool from IDT and amplified with PCR by first annealing at a 1:1 equimolar ratio, mixing 1 μl DNA template, 0.4 μl 100 µM oBW138, 0.4 μl 100 µM oBW139, 10 μl nuclease-free water, 12.5 μl 2× Q5 High Fidelity master mix and using the following temperature programme: 98 °C for 30 s, 35 cycles of 98 °C for 10 s, 65 °C for 30 s, 72 °C for 45 s, a final incubation at 72 °C for 5 min and hold at 4 °C. Following PCR, the DNA templates were purified using a 1.2× SPRI and used for in vitro transcription. Guide RNAs were transcribed using the NEB HiScribe kit (NEB E2040S) by mixing 100 ng of DNA template, 2 μl of 10× reaction buffer, 2 μl 100 mM ATP, 2 μl 100 mM GTP, 2 μl 100 mM CTP, 2 μl 100 mM UTP, 2 μl T7 RNA polymerase mix and nuclease-free water up to 20 μl, and incubated overnight at 37 °C.

Following an overnight in vitro transcription, DNA template was digested by adding 3 μl 10× DNase buffer, 2 μl DNase I and incubating for an additional 15 min at 37 °C. Guide RNAs were purified using a 2× SPRI reaction and checked for purity by running on a 15% TBE-urea gel (Invitrogen, EC6885BOX). Guide RNA concentration was quantified using the Broad Range RNA Qubit kit (Thermo Fisher, Q10210).

To perform Cas9-based depletion in our most-optimized condition, 2 ng of library was mixed with 1.5 μl NEB 3.1 buffer and sgRNA and NEB Cas9 at a 20,000:3,000:1 ratio of sgRNA:Cas9:DNA. The reaction was incubated at 37 °C for 2 h, after which Cas9 was stripped from the DNA by adding in 1 μl Proteinase K and 1 μl 10% SDS, and incubating for 15 min at 50 °C. The DNA library was purified with a 1.2× SPRI, eluted in 25 μl nuclease-free water and mixed with 25 μl 2× Q5 High Fidelity master mix, 0.4 μl 100 µM oBW170 and 0.4 μl 100 µM of a unique P7 index primer. The reactions were amplified with the following temperature programme: 98 °C for 30 s, 12 cycles of 98 °C for 10 s, 65 °C for 30 s, 72 °C for 30 s, a final incubation at 72 °C for 5 min and hold at 4 °C. Libraries were sequenced on the MiSeq reagent kit v.2 (300 cycles) (Illumina MS-102-2002) using the following read structure: 26 bp Read 1, 30 bp i5 index, 8 bp i7 index, 100 bp Read 2.

### Quantifying cell loading in the 10X microfluidic system

To quantify whether single bacterial cells could be loaded into the 10X microfluidic system, we first fixed 2 ml of *E. coli* MG1655 cells grown to OD = 0.4 overnight in 4 ml 4% formaldehyde. Cells were prepared as described above up to after the first wash following permeabilization. Following the first wash, cells were incubated in 50 μl 5 µM Sytox Green (Thermo Fisher, S7020) for 15 min. After the incubation, cells were washed twice in 100 μl of PBS-RI and then resuspended in 100 μl of 0.5× PBS-RI. Cells were counted and then loaded onto the 10X microfluidic system using the Chip A 5’ kit.

Following droplet generation, 5 μl of the mixture was transferred onto a glass coverslip and imaged on a Nikon TiE microscope with a Plan Apo ×20 objective and Hanamatsu ORCAFlash4.0 camera. Cells in each droplet were then manually counted for quantification.

### Plaque assays

To test the titre of phage preparations, 3 μl of phage was spotted in 10-fold serial dilutions on a lawn of *E. coli* MG1655 grown on 0.2% LB top agar with or without magnesium.

### Data preprocessing

Raw base calls were retrieved from the NovaSeq and processed with a custom version of Picard tools v.2.19.2 following the pipeline described in the original SciFi-seq pipeline^16^. Reads were aligned to a combination of one or more of *B. subtilis* 168, *E. coli* MG1655 and *E. coli* Nissle genomes using STAR (v.2.76)^47^ and annotated with featureCounts (v.2.0.0)^48^. Reads were filtered such that all the reads used for downstream analysis had mapQ score > 1, which correspond to reads that have aligned to 3 or less locations and mapped lengths greater than 20 bp. Annotated and filtered reads were loaded into Python 3.7.6, where custom code was written to assign non-rRNA reads to combinations of droplet and plate barcodes in pandas.

After assigning reads to barcode combinations, we filtered out ‘cell clumps’, which we defined as droplet barcodes in which a given droplet barcode had more than 8 associated plate barcodes. We split barcode combinations by condition (round-one barcodes) and performed another filtering step using the knee method for each condition^5,9^. We note that this step is important because bacteria in different conditions have different amounts of mean mRNA expression. When necessary, index collision rates were calculated by computing the fraction of cells with <85% of UMIs assigned to one species and then corrected to account for within-species interactions by multiplying a scale factor of $\frac{1}{2{pq}}$, where *p* is the frequency of species 1 and *q* the frequency of species 2, such that *p* + *q* = 1. After the last filtering step, a cell/gene matrix was made where the entries of the matrix are the number of UMIs that we measured for that gene in a particular cell.

### Cell identity determination

In cases where two species were processed with the same round-one barcode, barcode combinations were assigned to a specific species if >85% of UMIs mapped to unique species-specific transcripts. Otherwise, cells were designated as mixed.

### Single-cell analysis

Metrics for the scRNA-seq results were compiled and plotted using custom scripts in Python 3.7.6. Downstream analysis of single-cell data was performed using pipelines detailed in Seurat (v4.0.3)^49^. Data were first preprocessed by filtering out genes that were expressed in less than 10 cells and cells that expressed less than 10 UMIs. The data were then normalized by dividing the UMI counts in each cell by the total number of UMIs measured in that cell, multiplying by a scale factor of 100, adding a count of 1 to each entry and then log-normalizing the scaled values^49^. The normalized expression data were then scaled to have mean 0 and unit variance, and dimensionally reduced using principal component analysis (PCA). When necessary, the kurtosis of each principal component was computed by taking the matrix of cells by principal component coordinates and then calling the ‘kurtosis’ function from the R package moments^50^.

Following PCA, we computed a uniform manifold approximation representation and a shared neighbour graph using the first 10 principal components. We performed graph-based clustering on the shared neighbour graph to identify clusters of gene expression programmes using the Louvain algorithm (algorithm 3 in Seurat 4.0.3). Marker genes for each cluster were computed using two-sided Wilcoxon rank-sum test and corrected using Bonferroni correction. Further data analysis and plotting were performed using custom scripts in R.

Gene set enrichment analyses were performed using topGo (2.48.0). Briefly, marker genes were determined using the FindMarkers function in Seurat, whereby we compared the within-cluster average expression to out-of-cluster average expression and filtering for genes with *P* value < 0.05 (two-sided Wilcoxon rank-sum test). This list was then split into genes that were upregulated in the cluster and genes that were downregulated. The two lists of genes were then used for biological process term enrichment using two-sided Fisher’s exact test, in which the input was a vector of length (number of genes in the genome), and each entry in the vector was 1 if the index corresponded to a gene in the list of upregulated/downregulated (depending on whether we were testing up- or downregulated genes) genes and 0 otherwise. Following the test, the *P* values were −log_10_ transformed such that the most strongly enriched biological processes have the highest score. Selected processes to be plotted were those with the lowest *P* values after thresholding at 0.05.

To compute silhouette scores, we took the PCA matrix and cluster outputs from Seurat, and used the silhouette score function from the KBET package^51^.

### Comparison with bulk RNA-seq

Bulk RNA-seq data for exponentially growing *E. coli* were created following library construction methods as performed for M3-seq. Raw reads from the bulk data were aligned to the *E. coli* MG1655 genome and annotated as described above. Single-cell and bulk transcriptomes of exponentially growing *E. coli* were compared by computing the Pearson correlation of log_10_-normalized UMI count of each gene between the two measurements. Normalized UMI count for each gene in single-cell data was then computed by adding a pseudocount of 1 to each gene, summing over the UMI counts for that gene across all cells, dividing by the sum of total UMIs and multiplying by a scale factor. Normalized UMI counts for bulk measurements were computed as described above. The normalized UMI counts of the bulk and single-cell datasets were log_10_ transformed and used for plotting and correlation measurements.

### Marker gene identification

Marker genes for each cluster were defined as those observed in at least 5% of cells in that cluster and with the lowest adjusted *P* values (two-sided Wilcoxon rank-sum test) after thresholding to select genes with >0.5 log_2_ fold change between within-cluster and out-of-cluster average expression. For panels that plotted marker gene expression across clusters, a maximum of 6 genes were included per cluster.

### Testing for BC1-specific bias in clustering analysis

To identify potential clustering biases that could be driven by different BC1s, we computed a normalized cluster percentage for each cluster and BC1. The normalized cluster percentage was defined as: $\frac{p({B}_{i},\,{C}_{j})}{p({B}_{i})}$, where $p({B}_{i},\,{C}_{j})$ represents the fraction of cells in cluster *j* that have BC1 *i* and *B*_*i*_ the total fraction of cells in the population with BC1 *i*.

### Statistics and reproducibility

#### Experimental replicates

Unless otherwise stated, all representative images and micrographs were collected over a single set of acquired images. In Fig. 2g, experiments were repeated 3 times with similar results. Data from Figs. 2i,j,l, 4j,k and 5f were from a single set of acquired images (*N* = *1*).

#### Boxplot limits

Unless otherwise stated, within the boxplots the centre line represents the median, the lower and upper bounds of the box the 25th and 75th percentiles, respectively, and the limits of the whiskers 1.5× the distance from the 25th and the 75th percentiles.

### Reporting summary

Further information on research design is available in the Nature Portfolio Reporting Summary linked to this article.

### Supplementary information

Supplementary Information Legends for Supplementary Tables 1–4, and Videos 1 and 2.

Reporting Summary

Supplementary Video 1 Representative movie of the acid-stress recovery assay (Fig. 2g) conducted with *E. coli* MG1655 transformed with P_*gadB*_-*gfp*. Briefly, cells were treated with acid (pH 3.0) in early stationary phase for 1 h and then transferred to a fresh LB pad for imaging. GFP and phase channels are overlaid.

Supplementary Video 2 Representative movie of *E. coli* MG1655 transformed with P_*gadB*_-*gfp* during acid treatment (pH 3.0). Briefly, cells were grown to early stationary phase, transferred to an acidic pad (pH 3.0) and imaged over time. Indicating no increase in *gad* protein expression, GFP fluorescence steadily decreased during acid treatment. GFP and phase channels are overlaid.

Supplementary Tables 1–4

## Figures and Tables

**Extended Data Table 1:**
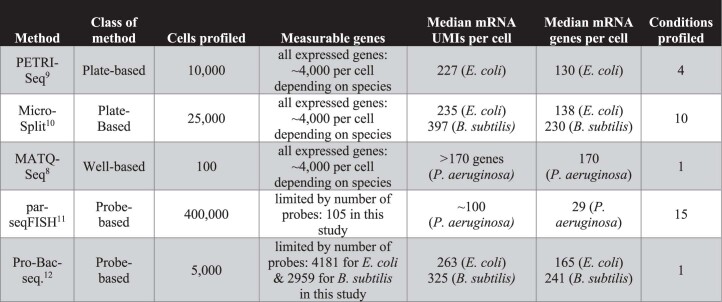
Statistics of existing bacterial scRNA-seq methods

Individual run statistics from previous studies of single-cell RNA-sequencing in bacteria. Numbers in each category were selected by taking maximum reported values. ‘Cells profiled’ indicates the maximum number of bacterial cells (for any number of strains) used in a single experiment. While ultimately the limiting factor is cost per cell, all else being equal, removing rRNA reads, either by using probes or rRNA depletion, will be more cost effective, with the latter also allowing for observation of unbiased gene sets. ‘Measurable genes’ indicates the number of genes that could theoretically be measured using the indicated method. ‘Median mRNA UMIs per cell’ indicates the maximum number of UMIs per mRNA per cell (for any number of strains and conditions) reported in the indicated study. ‘Median mRNA genes per cell’ same as ‘Median mRNA UMIs per cell’ except for genes. ‘Conditions profiled’ refers to the maximum number of reported samples/conditions profiled in a single experiment. While the limiting factor in conditions profiled is time and cost, we note that indexing multiple samples using indexed primers is currently the most time and cost efficient approach to profile multiple conditions.

## Data Availability

Sequencing data have been deposited to GEO (accession number GSE231935); raw image files have been uploaded in Zenodo (10.5281/zenodo.8168551) and are also available upon request.

## Code availability

All analysis and demultiplexing scripts are available at https://github.com/brwaang55/m3seq_scripts.
