# Supplementary material for: Single-cell massively-parallel multiplexed microbial sequencing (M3-seq) identifies rare bacterial populations and profiles phage infection
Source: Nat Microbiol. 2023 Aug 31;8(10):1846–62. doi: 10.1038/s41564-023-01462-3 (PMC10522482; doi:10.1038/s41564-023-01462-3)
Supplement: Supplementary file 1 — Legends for Supplementary Tables 1–4, and Videos 1 and 2. [file 41564_2023_1462_MOESM1_ESM.pdf]

nature microbiology

Article https://doi.org/10.1038/s41564-023-01462-3

Single-cell massively-parallel multiplexed microbial sequencing (M3-seq) identifies rare bacterial populations and profiles phage infection

In the format provided by the authors and unedited

Supplementary Table S1. Details of the single-cell sequencing experiments presented in this study. Statistics of the single-cell sequencing experiments presented in this study. "Cells loaded" refers to the number of single cells loaded into one lane of the Chromium Controller. "Single-cell transcriptomes recovered" were the number of single-cell transcriptomes that passed the UMI threshold (*Materials and Methods*). "Species" refers to the species analyzed in each experiment. eBW4 also included *P. aeruginosa* PA14 and *S. aureus* MRSA but cell / UMI recovery was low and reads mapping to these species were discarded. "Library treatment" refers to additional steps performed during library preparation.

Supplementary Table S2. Sample statistics from all experiments included in study. eBW4 also included *P. aeruginosa* PA14 and *S. aureus* MRSA but cell / UMI recovery was low and reads mapping to these species were discarded.

Supplementary Table S3. Oligonucleotides used in this study. Due to the large number of oligonucleotides used in this study, each workbook is split into the different purposes that the oligonucleotide was used for, which are "RT oligos", "rRNA depletion oligos", and "General oligos".

Supplementary Table S4. Marker genes for all the clusters and samples analyzed in this study. Each workbook here refers to marker genes for a single species (*E. coli* MG1655, *E. coli* Nissle, *B. subtilis* 168) within all the conditions profiled within that single-cell sequencing experiment (eBW1, eBW2, eBW3, eBW4). *p* values were adjusted for multiple comparisons using the Bonferroni correction.

Supplementary Video S1. Movie of *E. coli* MG1655 recovering from the acid-stress recovery assay. Representative movie of the acid-stress recovery assay (Fig. 2G) conducted with *E. coli* MG1655 transformed with P_{gadB}-*gfp*. Briefly, cells were treated with acid (pH 3.0) in early stationary phase for one hour and then transferred to a fresh LB pad for imaging. GFP and phase channels are overlaid.

Supplementary Video S2. Movie of *E. coli* MG1655 during strong acid treatment.
Representative movie of *E. coli* MG1655 transformed with P_{gadB}-*gfp* during acid treatment (pH 3.0). Briefly, cells were grown to early stationary phase, transferred to an acidic pad (pH 3.0), and imaged over time. Indicating no increase in *gad* protein expression, GFP fluorescence steadily decreased during acid treatment. GFP and phase channels are overlaid.
