# Supplementary material for: Single-cell massively-parallel multiplexed microbial sequencing (M3-seq) identifies rare bacterial populations and profiles phage infection
Source: Nat Microbiol. 2023 Aug 31;8(10):1846–62. doi: 10.1038/s41564-023-01462-3 (PMC10522482; doi:10.1038/s41564-023-01462-3)
Supplement: Supplementary file 2 — Reporting Summary [file 41564_2023_1462_MOESM2_ESM.pdf]

nature portfolio

Corresponding author(s): Ned Wingreen, Britt Adamson, Zemer Gitai

Last updated by author(s): Jul 20, 2023

Reporting Summary

Nature Portfolio wishes to improve the reproducibility of the work that we publish. This form provides structure for consistency and transparency in reporting. For further information on Nature Portfolio policies, see our Editorial Policies and the Editorial Policy Checklist.

Statistics

For all statistical analyses, confirm that the following items are present in the figure legend, table legend, main text, or Methods section.

n/a	Confirmed	
☐	☒	The exact sample size (*n*) for each experimental group/condition, given as a discrete number and unit of measurement
☐	☒	A statement on whether measurements were taken from distinct samples or whether the same sample was measured repeatedly
☐	☒	The statistical test(s) used AND whether they are one- or two-sided<br>*Only common tests should be described solely by name; describe more complex techniques in the Methods section.*
☒	☐	A description of all covariates tested
☐	☒	A description of any assumptions or corrections, such as tests of normality and adjustment for multiple comparisons
☐	☒	A full description of the statistical parameters including central tendency (e.g. means) or other basic estimates (e.g. regression coefficient) AND variation (e.g. standard deviation) or associated estimates of uncertainty (e.g. confidence intervals)
☐	☒	For null hypothesis testing, the test statistic (e.g. *F*, *t*, *r*) with confidence intervals, effect sizes, degrees of freedom and *P* value noted<br>*Give P values as exact values whenever suitable.*
☒	☐	For Bayesian analysis, information on the choice of priors and Markov chain Monte Carlo settings
☒	☐	For hierarchical and complex designs, identification of the appropriate level for tests and full reporting of outcomes
☐	☒	Estimates of effect sizes (e.g. Cohen's *d*, Pearson's *r*), indicating how they were calculated

Our web collection on statistics for biologists contains articles on many of the points above.

Software and code

Policy information about availability of computer code

Data collection	Sequencing was performed on the NovaSeq6000 at the Genomics Core Facility at Princeton University. Nikon NIS Elements Imaging Software (Version 4.6) was used to acquire images.
Data analysis	All custom code is located at https://github.com/brwaang55/m3seq_scripts.<br>DeLTA 2.0.0 was used to segment cells, Seurat 4.03 was used to perform single-cell analyses, topGo 2.48.0 was used to perform gene set enrichment analyses, kurtosis calculations were computed using the R package moments 0.14.1, and custom code was written in Python 3.7.6 or R 4.0.3. We used picard tools 2.19.2 to process reads, STAR2.7.6 to align our reads, and featureCounts 2.0.0 to annotate the reads. Further custom software was taken from the https://github.com/epigen/scifiRNA-seq.

For manuscripts utilizing custom algorithms or software that are central to the research but not yet described in published literature, software must be made available to editors and reviewers. We strongly encourage code deposition in a community repository (e.g. GitHub). See the Nature Portfolio guidelines for submitting code & software for further information.

Data

Policy information about availability of data

All manuscripts must include a data availability statement. This statement should provide the following information, where applicable:

- Accession codes, unique identifiers, or web links for publicly available datasets
- A description of any restrictions on data availability
- For clinical datasets or third party data, please ensure that the statement adheres to our policy

Sequencing data has been deposited to GEO (accession number GSE231935); raw image files are in the process of being uploaded to Zenodo (DOI 10.5281/zenodo.8168551), and is currently available upon request.Sequencing data has been deposited to GEO (accession number GSE231935); raw image files are in the process of being uploaded to Zenodo (DOI 10.5281/zenodo.8168551), and is currently available upon request.

Human research participants

Policy information about studies involving human research participants and Sex and Gender in Research.

Reporting on sex and gender	n/a
Population characteristics	n/a
Recruitment	n/a
Ethics oversight	n/a

Note that full information on the approval of the study protocol must also be provided in the manuscript.

Field-specific reporting

Please select the one below that is the best fit for your research. If you are not sure, read the appropriate sections before making your selection.

☒ Life sciences ☐ Behavioural & social sciences ☐ Ecological, evolutionary & environmental sciences

For a reference copy of the document with all sections, see nature.com/documents/nr-reporting-summary-flat.pdf

Life sciences study design

All studies must disclose on these points even when the disclosure is negative.

Sample size	Number of cells to load in S2A were chosen to match the range in Datlinger et. al. Number of cells was chosen in each experiment was chosen to minimize index collision rates from the calculations performed in Extended Data Figure 2c For imaging experiments, we picked a minimum 10 fields per view within each sample to ensure that there were no positional effects of the imaging system.<br><br>For imaging experiments, at least 10 fields of view were acquired. N=3 independent replicates were acquired for quantification of gad subpopulation (Fig. 2G). For all other imaging experiments, no sample size calculations were performed, sample size N=1 for qualitative confirmation of phenotypes observed in sequencing.
Data exclusions	We excluded droplet indices which had >8 associated plate barcodes in experiments where we used 96 round-one indices, and excluded droplet indices which had >14 round-one indices in experiments using a total of 288 round one indices. This was done to remove "cell clumps" which may have arosen during the experimental protocol. During single cell data preprocessing, we removed index combinations from each condition using the "knee" method, whereby we thresholded the number of UMIs/cell from the species by plotting the distribution of UMIs/cell. Data from Pseudomonas aeruginosa and Staphylococcus aureus were excluded from the final analyses in BW4 because the median UMIs/cell too low (<30/cell in Pseudomonas, <10/cell in Staphylococcus), which would not have allowed us to make reliable conclusions based off the data.
Replication	Two biological replicates of ciprofloxacin treated cells from E. coli MG1655, E. coli Nissle, and B. subtilis 168 were single cell sequenced to ensure that the single cell protocol reproduced the biological signature from two replicate samples treated with the same drug. For growth curve analyses, we performed 3 replicates to ensure reliability of the overexpression.
Randomization	No humans/animals were used. Control/treated samples came from the same original bacterial culture.
Blinding	No blinding was performed. Unsupervised clustering identified subpopulations before followup experiments.

Reporting for specific materials, systems and methods

We require information from authors about some types of materials, experimental systems and methods used in many studies. Here, indicate whether each material, system or method listed is relevant to your study. If you are not sure if a list item applies to your research, read the appropriate section before selecting a response.

Materials & experimental systems

n/a	Involved in the study	
☒	☐	Antibodies
☒	☐	Eukaryotic cell lines
☒	☐	Palaeontology and archaeology
☒	☐	Animals and other organisms
☒	☐	Clinical data
☒	☐	Dual use research of concern

Methods

n/a	Involved in the study	
☒	☐	ChIP-seq
☒	☐	Flow cytometry
☒	☐	MRI-based neuroimaging
